# Two colistin resistance-producing *Aeromonas* strains, isolated from coastal waters in Zhejiang, China: characteristics, multi-drug resistance and pathogenicity

**DOI:** 10.3389/fmicb.2024.1401802

**Published:** 2024-07-31

**Authors:** Hong-Xian Chen, Fang-Jie Chen, Qian-Jin Zhou, Shi-Lin Shang, Biao Tang, Zhong-Jie Xu, Li-Jun Duan, Jing-Lei Jin, Gui-Zong Xu, Mao-Cang Yan, Jiong Chen

**Affiliations:** ^1^State Key Laboratory for Managing Biotic and Chemical Threats to the Quality and Safety of Agro-Products, Ningbo University, Ningbo, China; ^2^School of Marine Sciences, Ningbo University, Ningbo, China; ^3^Key Laboratory of Aquacultural Biotechnology Ministry of Education, Ningbo University, Ningbo, China; ^4^State Key Laboratory for Managing Biotic and Chemical Threats to the Quality and Safety of Agro-Products, Zhejiang Academy of Agricultural Sciences, Hangzhou, China; ^5^Ningbo Haishu District Animal Husbandry and Veterinary Medicine Technical Management Service Station, Ningbo, China; ^6^Zhejiang Key Laboratory of Exploitation and Preservation of Coastal Bio-Resource, Zhejiang Mariculture Research Institute, Wenzhou, China

**Keywords:** *Aeromonas*, antibiotic resistance genes, mobile genetic elements, *mcr-3*, pathogenicity, coastal water

## Abstract

**Introduction:**

*Aeromonas* spp. are ubiquitous inhabitants of ecosystems, and many species are opportunistically pathogenic to humans and animals. Multidrug-resistant (MDR) *Aeromonas* species have been widely detected in hospitals, urban rivers, livestock, and aquatic animals.

**Results:**

In this study, we identified two *Aeromonas* isolates, namely *Aeromonas veronii* 0728Q8Av and *Aeromonas caviae* 1029Y16Ac, from coastal waters in Zhejiang, China. Both isolates exhibited typical biochemical characteristics and conferred MDR to 11 kinds of antibiotics, remaining susceptible to ceftazidime. Whole-genome sequencing revealed that both isolates harbored multiple antibiotic resistance genes (ARGs) and several mobile genetic elements (MGEs) on the chromosomes, each containing a resistance genomic island (GI), a typical class 1 integron, a transposon, and various insertion sequences (ISs). Most ARGs were situated within the multiple resistance GI, which contained a class 1 integron and a transposon in both *Aeromonas* isolates. Furthermore, a chromosomal *mcr-3.16* gene was identified in *A. veronii* 0728Q8Av, while a chromosomal *mcr-3.3* was found in *A. caviae* 1029Y16Ac. Both mcr-3 variants were not located within but were distanced from the multidrug resistance GI on the chromosome, flanking by multiple ISs. In addition, a *mcr-3-like* was found adjacent to *mcr-3.16* to form a tandem *mcr-3.16*-*mcr-3-like*-*dgkA* structure; yet, *Escherichia coli* carrying the recombinants of *mcr-3-like* did not exhibit resistance to colistin. And an incomplete *mcr-3-like* was found adjacent to *mcr-3.3* in *A. caviae* 1029Y16Ac, suggesting the possibility that *mcr-3* variants originated from *Aeromonas* species. *In vivo* bacterial pathogenicity test indicated that *A. veronii* 0728Q8Av exhibited moderate pathogenicity towards infected ayu, while *A. caviae* 1029Y16Ac was non-virulent.

**Discussion:**

Thus, both *Aeromonas* species deserve further attention regarding their antimicrobial resistance and pathogenicity.

## 1 Introduction

Antibiotic resistance genes (ARGs) are considered emerging environmental contaminants due to their potential risks to human and animal health (Zheng et al., 2021). Infections caused by multidrug-resistant (MDR) Gram-negative bacteria (GNB) are increasingly common and pose a paramount therapeutic challenge, raising significant concerns worldwide (Osei Sekyere et al., 2016; Vivas et al., 2019). Colistin is considered one of the few “last-resort” antibiotics against MDR GNB infections (Osei Sekyere et al., 2016; Yin et al., 2017). The emergence and rapid dissemination of the mobile colistin resistance (*mcr*) genes further compound the challenges in antibiotic treatment of MDR bacteria (Vivas et al., 2019; Shen et al., 2020).

*Aeromonas*, a Gram-negative bacillus, is widely distributed in the environment, particularly in freshwater and estuarine ecosystems (Janda and Abbott, 2010). Currently, the *Aeromonas* genus encompasses 36 species, many of which are opportunistically pathogenic to both animals and humans (Janda and Abbott, 2010; Fernández-Bravo and Figueras, 2020). *Aeromonas* spp. infect humans, causing various gastroenteritis and extra-intestinal diseases (Fernández-Bravo and Figueras, 2020; Carusi et al., 2023). Long recognized as a significant pathogen in aquatic animals, *Aeromonas* instigates a broad spectrum of opportunistic infections, resulting in severe clinical manifestations such as sepsis, bleeding, ulcers, ascites, and high mortality (Janda and Abbott, 2010; Carusi et al., 2023). Many studies have demonstrated that *Aeromonas* serves as a MDR carrier and a reservoir of many ARGs like *mar* genes (Piotrowska and Popowska, 2015; Ling et al., 2017; Eichhorn et al., 2018; Shen et al., 2020).

The coastal waters, being significant areas of human activity, harbor a wealth of ARGs and antibiotic-resistant bacteria (ARB) (Zhu et al., 2017; Zheng et al., 2021). These genes and bacteria originate not only from coastal aquaculture but also from various human activities such as animal husbandry, freshwater aquaculture, clinical medicine, and industrial production (Zhu et al., 2017; Jang et al., 2018; Zheng et al., 2021). They are introduced into coastal water environments through mechanisms such as rainfall and sewage discharge (Shao et al., 2018). Exposure of environmental microorganisms to this mixture stimulates horizontal gene transfer (HGT) events, spreading genetic resistance elements across different microbial strains and increasing microbial abundance and penetration in new hosts (Juhas et al., 2009; Shao et al., 2018). *Aeromonas* spp. is ubiquitously distributed in coastal waters and mariculture animals, many of which harbors abundant ARGs and mobile genetic elements (MGEs) (Piotrowska and Popowska, 2014; Carusi et al., 2023). Although *Aeromonas* carrying *mcr* genes have been repeatedly detected in freshwater animals and water bodies (Eichhorn et al., 2018; Xu et al., 2020; Sakulworakan et al., 2021), the incidence of mobile colistin resistance determinants and their genetic environment in *Aeromonas* genomes from coastal waters and marine animals have largely been ignored. In this study, we investigated the antibiotic susceptibility of two MDR *Aeromonas* species isolated from the coastal waters near Wenzhou, Zhejiang Province, China. Through genome sequencing, we examined the genetic profiles for antimicrobial resistance, analyzed the structural characteristics of MDR gene islands on their chromosomes, and investigated the distribution of *mcr* variants. Additionally, we experimentally tested the ability of the *mcr* variants to mediate colistin resistance. Furthermore, we assessed the *in vivo* pathogenicity of the two MDR *Aeromonas* species in ayu (*Plecoglossus altivelis*), an important amphidromous economic fish species found in East Asia.

## 2 Materials and methods

### 2.1 Bacterial strains

In our previous study, we selected 25 sampling sites in the coaster waters near Wenzhou in the East China Sea and collected surface seawater samples (Jin et al., 2021). We isolated culturable bacteria resistant to sulfonamides, tetracyclines, and quinolones from the seawater samples using antibiotic resistance plates and detected the target ARGs in these isolates using polymerase chain reaction (PCR). We isolated 1,605 bacterial strain with antibiotic resistance phenotypes, among which 51 isolates tested positive for resistance genes related to sulfonamides, tetracyclines, and quinolones, with 43 of them belonging to the genus *Aeromonas* (Jin et al., 2021). Additionally, we identified 39 MDR strains (Jin et al., 2021). Using PCR primers for the *mcr* genes, we identified 2 *Aeromonas* isolates containing the *mcr-3* variants (Jin et al., 2021; Xu et al., 2021) (Supplementary Table S1). Preliminary 16S rDNA sequencing suggested that both isolates likely belonged to *Aeromonas veronii* and *Aeromonas caviae*. In this study, further identification of both isolates was performed using multilocus phylogenetic analysis (MLPA) based on six genes (*gyrB*, *rpoD*, *dnaJ*, *gyrA*, *dnaX*, and *atpD*) (Martinez-Murcia et al., 2011). Phylogenetic trees were constructed based on the concatenated sequences containing these six genes using the maximum likelihood method in the MEGA 7.1 software package. Subsequently, biochemical tests were conducted on the isolates using biochemical test kits (Tiangen, Beijing, China).

### 2.2 Antimicrobial susceptibility testing

Antimicrobial susceptibility to 12 antibiotics belonging to seven antibiotic classes, including β-lactams [ampicillin (AMP) and ceftazidime (CAZ)], tetracyclines [oxytetracycline (OTC) and tetracycline (TET)], amphenicols [chloramphenicol (CHP) and florfenicol (FFC)], sulfonamides [sulfamethoxazole (SMZ) and trimethoprim (TMP)], quinolones [ciprofloxacin (CIP) and enrofloxacin (ENR)], aminoglycosides [gentamycin (GEN)], and polymyxin [colistin (COL)], was assessed using the disc diffusion method, with modifications derived from the methodology outlined by Huq (2020). Briefly, *A. veronii* 0728Q8Av and *A. caviae* 1029Y16Ac were cultured overnight in Mueller Hinton II (cation-adjusted)/CAMHB broth (Solarbio, Beijing, China). A 0.1 mL bacterial culture of each strain was evenly spread on a Mueller–Hinton (MH) agar plate. The sterile paper discs (7 mm in diameter) containing the antibiotics (10 μg/disc for APM, GEN, 30 μg/disc for CAZ, TET, OTC, CHP, FFC, 5 μg/disc for CIP, ENR, TMP, 300 IU/disc for COL, 250 μg/disc for SMZ) were placed on the surface of the inoculated MH agar plates. The plates were then incubated at 28°C, and the inhibition zones were measured after 18 h of incubation.

The antibiotics that induced resistance in either of the tested bacterial strains were selected to calculate the minimum inhibitory concentrations (MICs) against both strains. This was achieved using the broth microdilution method (BMD) recommended by the Clinical and Laboratory Standards Institute (CLSI) (CLSI, 2018, M100-S28). BMD is conducted using Mueller Hinton II (cation-adjusted)/CAMHB broth, a range of 2-fold dilutions of antibiotics (ranging from 0.5 to 256 μg/mL), and a bacterial inoculum density was adjusted equivalent to a 0.5 McFarland standard per well. The MIC breakpoints were interpreted according to the European Committee on Antimicrobial Susceptibility Testing (EUCAST)^1^ and CLSI guidelines (CLSI, 2018, M100-S28). *Escherichia coli* ATCC 25922 was used as a quality control.

### 2.3 Whole-genome sequencing and sequence analysis

The genomic DNA of *A. veronii* 0728Q8Av and *A. caviae* 1029Y16Ac was extracted using cetyltrimethylammonium bromide method, following the manufacturer’s protocol BGI-PB-TQ-DNA-003A0. Subsequently, a 20 kb fragment library was constructed for each isolate, utilizing high-quality genomic DNA that met the requirements for whole-genome sequencing. Sequencing was carried out on a Pacbio Sequel II platform (BGI, Shenzhen, China). The reads were assembled using Canu (version 1.5). After multiple adjustments to achieve the optimal assembly, base correction, circularization, and plasmid library alignment were performed on the assembled sequences to obtain the final assembly results. Gene prediction was performed with Glimmer (version 3.02). The annotation of genes was accomplished through in-house pipeline on the RAST server^2^ (Ebu et al., 2023). Acquired antimicrobial resistance genes were predicted using ResFinder 4.1^3^ with a threshold of percent identity set at 90% and a minimum coverage length of 60%. Genomic islands (GIs) were determined using IslandPath-DIMOB^4^ (Bertelli and Brinkman, 2018). Insertion sequences (ISs) were identified through ISfinder^5^ (Siguier et al., 2006). Transposons and integrons were predicted using TnCentral^6^ and Integron Finder,^7^ respectively (Damas et al., 2022). Comparative analysis of genome sequences was performed using Easyfig with a maximum *e*-value of 0.001 and identification threshold set at 98% and BRIG (with an identification threshold of 50%) (Tang et al., 2022). Virulence factors were predicted using the Virulence Factor Database (VFDB, http://www.mgc.ac.cn/VFs/) (Liu et al., 2022).

### 2.4 *mcr-3* variant functionality assay

According to Jin et al. (2021), the prevalence of *Aeromonas* spp. with *mcr* genes in coastal waters is low (2 out of 51). *A. veronii* 0728Q8Av and *A. caviae* 1029Y16Ac show different levels of resistance to colistin and harbor different *mcr-3* variants. To assess whether these *mcr-3* variants affect resistance to colistin, we conducted functionality assays. To *in silico* verify *mcr-3* variants in both *A. veronii* 0728Q8Av and *A. caviae* 1029Y16Ac, the sequences of *mcr-3.16*, *mcr-3.3*, and *mcr-3-like*, was aligned, respectively, referred to sequences of *mcr-3* variants downloaded from NCBI.^8^ And a phylogenetic tree was constructed based on *mcr-3* variants’ sequences using the Maximum likelihood method in the MEGA 7.1 software package.

To determine the function of *mcr-3* variants, four DNA fragments, corresponding to each open reading frame (ORF) of *mcr-3.16*, *mcr-3-like*, *mcr-3.16-mcr-3-like*, and *mcr-3.3* regions along with their 5′- and 3′-flanking regions, respectively, were amplified using the primers Q8MCR3.16-F/Q8MCR3.16-R, Q8MCR-3-like-F/Q8MCR-3-like-R, Q8MCR3.16-F/MCR-3-like-R, and Y16MCR3.3-F/Y16MCR3.3-R, and cloned into pUC19 vector by digestion of *Bam*HI and *Eco*RI, respectively. The recombinant plasmids were separately transformed into *E. coli* DH5α. Transformants were screened on LB agar plates containing 50 μg/mL ampicillin.

Meanwhile, the ORFs of *mcr-3.16*, *mcr-3-like*, *mcr-3.16-mcr-3-like*, and *mcr-3.3*, were amplified using the primers Q8MCR3.16-BMQ-F/Q8MCR3.16-BMQ-R, Q8MCR-3-likeBMQ-F/Q8MCR-3-likeBMQ-R, Q8MCR3.16-BMQ-F/Q8MCR-3-likeBMQ-R, and Y16MCR3.3-BMQ-F/Y16MCR3.3-BMQ-R, and cloned into pET28a vector by digestion of *Xho*I and *Eco*RI, respectively. The recombinant plasmids were separately transformed into *E. coli* BL21. Transformants were screened using 50 μg/mL kanamycin and were induced by addition of IPTG. Colistin susceptibility of *E. coli* transformed with any of the recombinant plasmids was determined in triplicates.

To determine whether the *mcr-3* variants in *A. veronii* 0728Q8Av and *A. caviae* 1029Y16Ac are located on the chromosome or plasmid, conjugation assays were conducted as described previously (Sun et al., 2016; Tang et al., 2024). The *E. coli* J53 was used as the recipient strain, and *A. veronii* 0728Q8Av or *A. caviae* 1029Y16Ac was the donor strain. The *E. coli* ECCNB20-2 carrying *mcr-1* was used as a positive control donor (Chang et al., 2020). All bacterial strains were cultured to be in the logarithmic growth phase in LB broth at 37°C. Each of the donor bacteria, that is *A. veronii* 0728Q8Av, *A. caviae* 1029Y16Ac, and *E. coli* ECCNB20-2, was mixed with *E. coli* J53 in suitable proportion, and the bacterial mixture was transferred to filter paper on the LB agar and cultured for 8 h. Then the filter paper was suspended into a centrifuge tube containing LB broth and serially diluted. Each diluted bacterial solution (10 μL) was cultured onto screening LB agar supplemented with 2 μg/mL colistin and 100 μg/mL NaN_3_, and with 100 μg/mL NaN_3_, respectively. Conjugation transfer was determined as described by Tang et al. (2022).

### 2.5 Bacterial pathogenicity assessment

Healthy ayu, weighing 20–25 g, were purchased from a commercial farm in Ningbo, China. Fish were raised in a recirculating system with filtered water at 20–22°C (Zhou et al., 2020). The fish were fed with pelleted dry food once a day and acclimatized to laboratory conditions for 2 weeks before experiments. Then, randomly allocate the fish into 21 tanks, each containing 10 individuals. The fish in each three tanks were intraperitoneally administered 100 μL of bacterial suspension of *A. veronii* 0728Q8Av and *A. caviae* 1029Y16Ac, at concentrations of 1 × 10^7^, 1 × 10^8^, and 1 × 10^9^ CFU/mL. Additionally, three tanks of fish were injected sterile PBS in volumes matching those of the experimental groups, serving as negative controls. All fish were kept at 20–22°C for 7 days to observe and record the morbidity and mortality rates. The degree of virulence, expressed as the 50% mean lethal dose (LD_50_), was calculated using the Bliss method (Finney, 1985). All intervention measures are strictly carried out in accordance with the guiding principles of the Animal Experiment Ethics Committee of Ningbo University (No. 11102).

### 2.6 Nucleotide sequence accession numbers

The complete genome sequences of *A. veronii* 0728Q8Av and *A. caviae* 1029Y16Ac were submitted to GenBank under Bioproject accession numbers PRJNA1070378 and PRJNA1070380, respectively.

## 3 Results

### 3.1 Characteristics of *Aeromonas veronii* 0728Q8Av and *Aeromonas caviae* 1029Y16Ac

**Figure 1:**
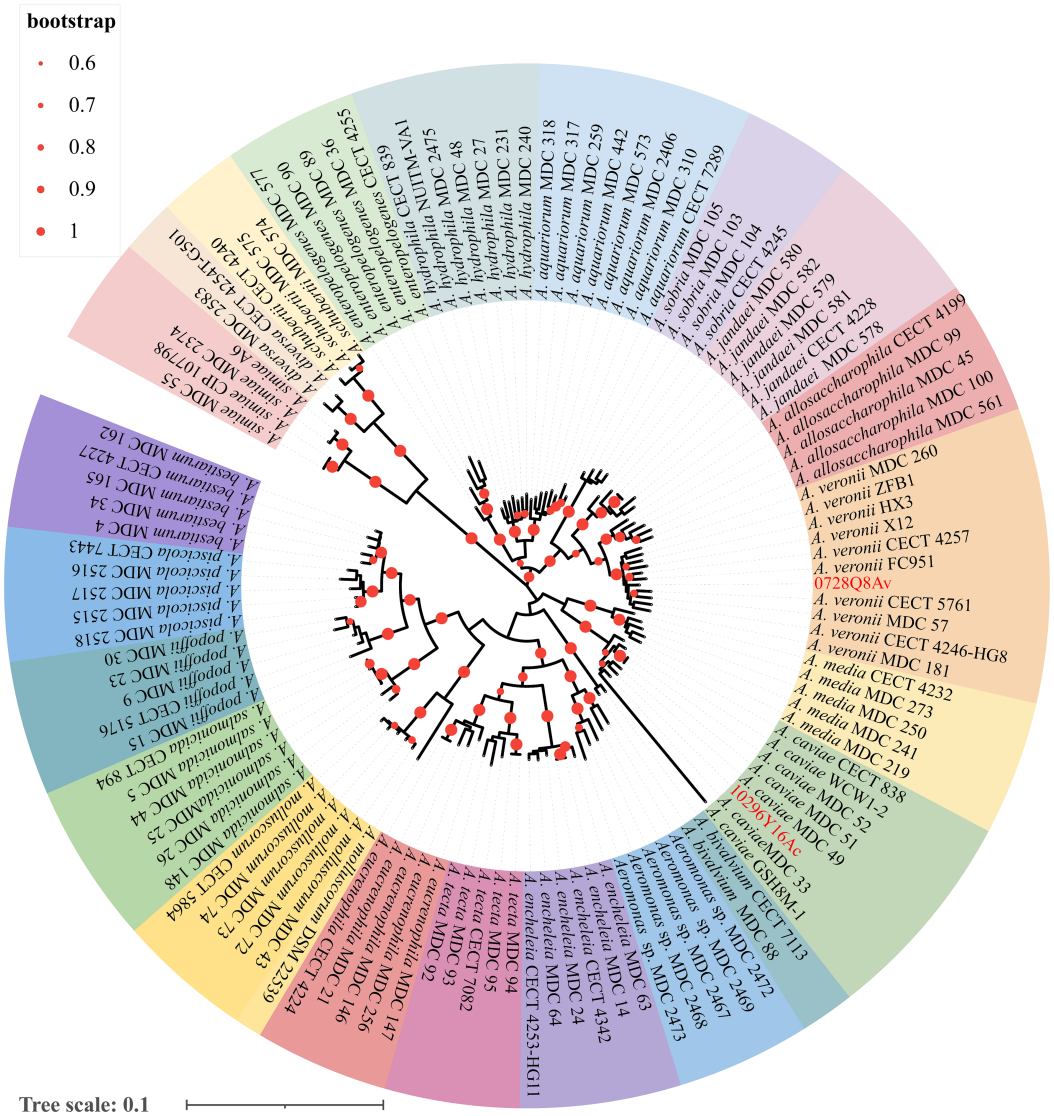
The multilocus phylogenetic tree analysis based on the sequences of six house-keeping genes (*gyrB*, *rpoD*, *dnaJ*, *gyrA*, *dnaX*, and *atpD*) from *Aeromonas* spp. using the maximum likelihood method. The values at the forks indicate the percentage of bootstrap values (1,000 replicates; shown only when >60%). Scale bar shows the number of substitutions per base. Accession numbers of the sequences used are listed in Supplementary Table S2.

Jin et al. (2021) identified both *mcr-3.16* and *mcr-3-like* in strain 0728Q8Av and *mcr-3.3* in strain 1029Y16Ac by DNA sequencing of PCR products. In this study, MLPA based on six house-keeping genes demonstrated that strain 0728Q8Av clustered together with selected *A. veronii* strains and strain 1029Y16Ac clustered together with *A. caviae* strains, which indicated that the two isolates belonged to *A. veronii* and *A. caviae*, respectively (Figure 1).

Regarding the biochemical characteristics, both *A. veronii* 0728Q8Av and *A. caviae* 1029Y16Ac exhibited numerous identical traits. For example, both isolates demonstrated growth at a concentration of 1–3% sodium chloride (NaCl) (w/v) but were unable to be maintained at >6% NaCl. They were both positive for acid formation when provided with glucose, sucrose, mannose, maltose, OPNG, mannitol, and saligenin, while showing negativity towards xylose, raffinose, and sorbose. Additionally, both bacterial isolates demonstrated the ability to produce oxidase, indole, and arginine dihydrolase (Table 1). However, the two bacterial isolates presented some differing characteristics. Specifically, *A. veronii* 0728Q8Av, unlike *A. caviae* 1029Y16Ac, tested positive in the Voges-Proskauer test and exhibited urea hydrolysis. *A. caviae* 1029Y16Ac could utilize arabinose and lactose, producing acid, whereas *A. veronii* 0728Q8Av could not (Table 1).

**Table 1:** Biochemical characteristics of both *A. veronii* 0728Q8Av and *A. caviae* 1029Y16Ac.

Characteristics		Reactions		Characteristics		Reactions	
		*A. veronii* 0728Q8Av	*A. caviae* 1029Y16Ac			*A. veronii* 0728Q8Av	*A. caviae* 1029Y16Ac
Hydrolysis of	Voges–Proskauer test	+	−	Produc-tion of	H_2_S	−	−
	Urea	+	−		Oxidase	+	+
Acid formation from	Glucose	+	+		Indole	+	+
	Sucrose	+	+		Arginine dihydrolase	+	+
	Mannose	+	+	Growth on	At 1% of NaCl	+	+
	Maltose	+	+		At 3% of NaCl	+	+
	Xylose	−	−		At 6% of NaCl	−	−
	Arabinose	−	+		At 8% of NaCl	−	−
	OPNG	+	+		At 10% of NaCl	−	−
	Raffinose	−	−				
	Lactose	−	+				
	Sorbose	−	−				
	Mannitol	+	+				
	Saligenin	+	+				

+, positive; −, negative.

### 3.2 Antibiotic susceptibility

The susceptibility testing showed that *A. veronii* 0728Q8Av exhibited resistance to COL (with a MIC of 8 μg/mL), OTC (64 μg/mL), TET (32 μg/mL), TMP (32 μg/mL), SMZ (256 μg/mL), AMP (64 μg/mL), CHP (16 μg/mL), and FFC (64 μg/mL), and intermediate to GEN (8 μg/mL), CIP (2 μg/mL), and ENR (2 μg/mL), but remained susceptible to CAZ (4 μg/mL) (Table 2). In contrast, *A. caviae* 1029Y16Ac exhibited resistance to COL (with a MIC of 4 μg/mL), AMP (32 μg/mL), TET (32 μg/mL), OTC (32 μg/mL), TMP (256 μg/mL), SMZ (256 μg/mL), CIP (4 μg/mL), ENR (4 μg/mL), and intermediate to CHP (8 μg/mL), FFC (16 μg/mL), and GEN (8 μg/mL), but remained susceptible to CAZ (8 μg/mL) (Table 2).

**Table 2:** Antibiotic resistance phenotype and antibiotic resistance genotypes of both *A. veronii* 0728Q8Av and *A. caviae* 1029Y16Ac.

Antibiotic type	Antibiotics used in antimicrobial susceptibility testing	R/I/S		ARGs		
		*A. veronii* 0728Q8Av	*A. caviae* 1029Y16Ac		*A. veronii* 0728Q8Av	*A. caviae* 1029Y16Ac
Polymyxin	Colistin (COL)	R	R	*mcr-3.16*	+	−
				*mcr-3.3*	−	+
				*mcr-3-like*	+	−
Quinolone	Ciprofloxacin (CIP)	I	R	*qnrVC4*	+	−
	Enrofloxacin (ENR)	I	R			
Sulfonamides	Sulfamethoxazole (SMZ)	R	R	*sul1*	+	+
	Trimethoprim (TMP)	R	R	*dfrA14*	+	−
Beta-lactam	Ampicillin (AMP)	R	R	*bla_OXA-10_*	+	+
	Ceftazidime (CAZ)	S	S			
	Not tested			*bla_VEB-3_*	−	+
				*bla_MOX-6_*	−	+
				*ampS*	+	−
				*cphA4*	+	−
Amphenicol	Chloramphenicol (CHP)	R	I	*catB3*	−	+
				*floR*	+	−
				*cmlA1*	+	−
	Florfenicol (FFC)	R	I	*floR*	+	−
				*cmlA1*	+	−
Tetracycline	Tetracycline (TET)	R	R	*tet(E)*	+	+
	Oxytetracycline (OTC)	R	R			
Aminoglycoside	Gentamicin (GEN)	I	I	*aac(6′)-Ib3*	+	+
	Not tested			*aph(3″)-Ib*	+	−
				*aph(6)-Id*	+	−
				*aadA1*	+	+

+, presence of the ARG; −, absence of the ARG; R, resistant; I, intermediate; S, susceptible, referenced from the Clinical and Laboratory Standards Institute (CLSI). Not tested: the corresponding antibiotic for this ARG was not tested in this experiment.

### 3.3 Genomic analysis of *Aeromonas veronii* 0728Q8Av and *Aeromonas caviae* 1029Y16Ac

*A. veronii* 0728Q8Av harbored one circular chromosomal genome of 4,752,946 bp with G + C content of 58.5% and no plasmid was found. *A. caviae* 1029Y16Ac harbored one circular chromosomal genome of 4,779,291 bp with G + C content of 60.86% and two plasmids, 1029Y16AcP1 (21,050 bp) and 1029Y16AcP2 (8,340 bp).

Acquired ARGs were identified by Resfinder 4.1 analysis. The chromosomal genome of *A. veronii* 0728Q8Av contained eight types of acquired ARGs indicating resistance to aminoglycoside [*aac(6′)-Ib3*, *aph(3″)-Ib*, *aph(6)-Id*, *aadA1*], polymyxin (*mcr-3.16*, *mcr-3-like*), quinolone (*qnrVC4*), sulfonamides (*sul1*, *dfrA14*), beta-lactam (*bla_OXA-10_*, *ampS*, *cphA4*), tetracycline [*tet(E)*], amphenicol (*floR*, *cmlA1*), and quaternary ammonium compound (*qacE∆1*). The genome contained 15 GIs and almost all the ARGs were located on the GI1 of *A. veronii* 0728Q8Av (AvGI1), except for *mcr-3.16*, *mcr-3-like*, *ampS* and *cphA4* (Supplementary Figure S1A; Supplementary Table S3). The chromosomal genome of *A. caviae* 1029Y16Ac contained seven types of acquired ARGs indicating resistance to aminoglycoside [*aac(6′)-Ib3*, *aadA1*], polymyxin (*mcr-3.3*), sulfonamides (*sul1*), beta-lactam (*bla_VEB-3_*, *bla_MOX-6_*, *bla_OXA-10_*), tetracycline [*tet(E)*], amphenicol (*catB3*) and quaternary ammonium compound (*qacE∆1*). No ARGs were identified on plasmid 1029Y16AcP1 and 1029Y16AcP2. The chromosomal genome presented 23 GIs, and almost all the ARGs were located on the GI11 of *A. caviae* 1029Y16Ac (AcGI11), except for *mcr-3.3*, *bla_MOX-6_*, and *tet(E)* (Table 2; Supplementary Figure S1B; Supplementary Table S3).

The virulence factors’ prediction suggested that virulence factors were significantly different between *A. veronii* 0728Q8Av and *A. caviae* 1029Y16Ac (Table 3). The Tap type IV pili of both strains exhibited similar structural features. However, *A. veronii* 0728Q8Av possessed 16 genes related to MSHA type IV pili, whereas *A. caviae* 1029Y16Ac had only 3. And *A. veronii* 0728Q8Av had type I and Flp type IV pili, whereas *A. caviae*1029Y16Ac did not. Flagella analysis revealed that both strains had polar flagella. However, *A. veronii* 0728Q8Av possessed 54 genes related to polar flagella, whereas *A. caviae* 1029Y16Ac had only 7. Both stains had a type II secretion system (T2SS) containing 14 genes. Toxin analysis revealed that both strains contained Hemolysin and Thermostable hemolysin (TH). Interestingly, *A. veronii* 0728Q8Av had Aerolysin, but *A. caviae* 1029Y16Ac did not.

**Table 3:** Main virulence factors for *A. veronii* 0728Q8Av and *A. caviae* 1029Y16A.

Types	Virulence factors	Strains		Gene count	
		*A. veronii* 0728Q8Av	*A. caviae* 1029Y16Ac	*A. veronii* 0728Q8Av	*A. caviae* 1029Y16Ac
Pili	Type I	+	−	7	−
	Flp type IV	+	−	13	−
	MSHA type IV	+	+	16	3
	Tap type IV	+	+	20	18
Flagella	Lateral flagella	−	−	−	−
	Polar flagella	+	+	54	7
Secretion system	T2SS	+	+	14	14
	T3SS	−	−	−	−
	T4SS	−	−	−	−
	T6SS	−	−	−	−
Toxin	Aerolysin	+	−	1 (*aerA*)	
	Hemolysin	+	+	1 (hlyA)	1 (hlyA)
	Thermostable hemolysin (TH)	+	+	1 (hemolysin-related hemolysin gene, *trh*)	1 (*trh*)

### 3.4 Functional identification in mediating colistin resistance of *mcr-3* variants

**Figure 2:**
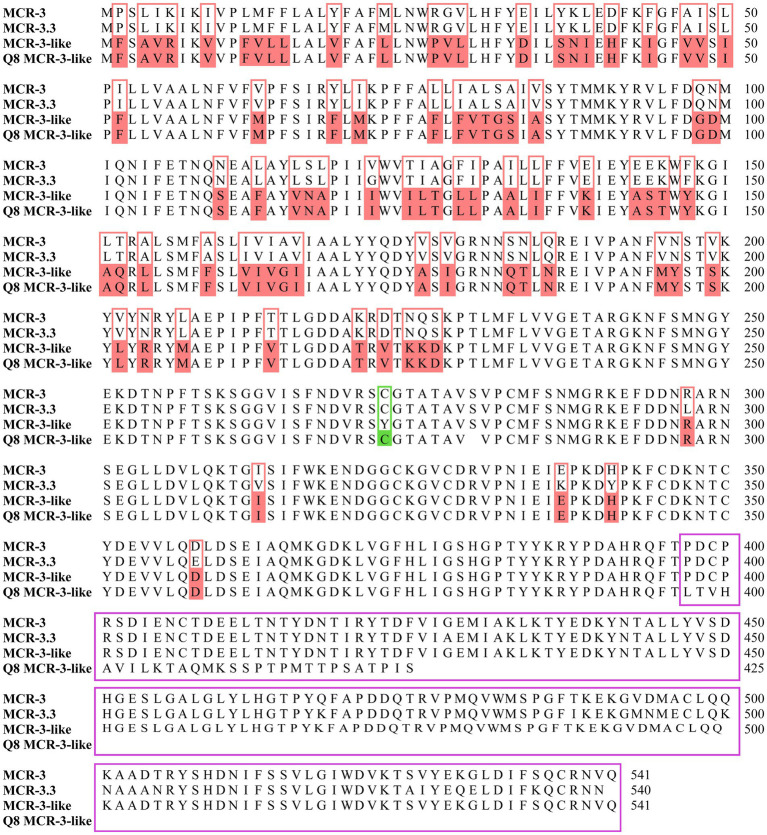
Alignment of the deduced amino acid sequences of Q8 MCR-3-like (*A. veronii* 0728Q8Av, PRJNA1070378) with previously reported MCR-3 (*E. coli* pWJ1, WP-039026394.1), MCR-3.3 (*Aeromonas veronii* 172, WP-099982814.1) and MCR-3-like (*Aeromonas veronii* 172, MF495680). The amino acid residues of Q8 MCR-3-like identical to MCR-3-like but differing from MCR-3 and MCR-3.3 were highlighted in pink boxes. The 273rd amino acid residue at the N-terminus of Q8 MCR-3-like differed from that of MCR-3-like but matched those of MCR-3 and MCR-3.3, highlighted in a green box. A 145-aa substitution by 29 amino acids at the C-terminus of Q8 MCR-3-like, compared to MCR-3-like, was indicated by a purple box.

Blast analysis revealed that both *mcr-3.16* (1623-bp of ORF) of *A. veronii* 0728Q8Av and *mcr-3.3* (1623-bp of ORF) of *A. caviae* 1029Y16Ac exhibited 100% identity to previous reported *mcr-3.16* (WP-111273845.1) and *mcr-3.3* (WP-099982814.1). MCR-3-like of *A. veronii* 0728Q8Av provided a 145-aa substitution by 29 amino acids at the carbon terminus, while a single amino acid difference occurred at the 273rd residue within the 396 amino acid residues at the Nitrogen terminus, compared to reported MCR-3-like (MF495680) (Figure 2). The phylogenetic tree unveiled a close clustering of both MCR-3.16 and MCR-3.3 with other predetermined MCR-3 variants. Additionally, the MCR-3-like variant in this study exhibited clustering alongside the one reported in *A. veronii* isolated from retail chicken (MF495680) (Supplementary Figure S2).

To determine the function in mediating colistin resistance of *mcr-3* variants, 2,685 bp, 1,920 bp, 4,008 bp, and 2,787 bp DNA fragments, corresponding to the ORFs of *mcr-3.16*, *mcr-3-like*, *mcr-3.16-mcr-3-like*, and *mcr-3.3* along with their 5′- and 3′-flanking regions, respectively, were amplified to construct the recombinants pUC19-*mcr-3.16*, pUC19-*mcr-3-like*, pUC19-*mcr-3.16-mcr-3-like*, and pUC19-*mcr-3.3*, and transformed into *E. coli* DH5α. The results showed that transformants containing pUC19-*mcr-3.16*, pUC19-*mcr-3.16-mcr-3-like*, and pUC19-*mcr-3.3*, had colistin MICs of 4 μg/mL, 4 μg/mL, and 2 μg/mL, respectively, which were 8-, 8- and 4-fold higher than the MIC of DH5α containing pUC19 alone (0.5 μg/mL). Transformants containing pUC19-*mcr-3-like* had a colistin MIC of 0.5 μg/mL, similar to DH5α containing pUC19 alone (Table 4). Meanwhile, the ORFs of the three *mcr-3* variants were cloned to construct the recombinants pET28a-*mcr-3.16*, pET28a-*mcr-3-like*, pET28a-*mcr-3.16-mcr-3-like*, and pET28a-*mcr-3.3*. The results showed that the transformants containing pET28a-*mcr-3.16*, pET28a-*mcr-3.16-mcr-3-like*, and pET28a-*mcr-3.3*, had colistin MICs of 16 μg/mL, which were 2-fold higher than the MIC of BL21 containing pET28a alone (8 μg/mL). Transformants containing pET28a-*mcr-3-like* had a colistin MIC of 8 μg/mL, similar to BL21 containing pET28a alone (Table 4).

**Table 4:** Colistin susceptibility profiles of *A. veronii* 0728Q8Av and *A. caviae* 1029Y16Ac harboring *mcr-3.16* and *mcr-3-like*, and *mcr-3.3*, respectively.

*E. coli* strain	MIC (μg/mL) of colistin
DH5α-pUC19	0.5
DH5α-pUC19-*mcr-3.16*	4
DH5α-pUC19-*mcr-3-like*	0.5
DH5α-pUC19-*mcr-3.16*-*mcr-3-like*	4
DH5α-pUC19-*mcr-3.3*	2
BL21-pET28a	8
BL21-pET28a-*mcr-3.16*	16
BL21-pET28a-*mcr-3*-like	8
BL21-pET28a-*mcr-3.16*-*mcr-3-like*	16
BL21-pET28a-*mcr-3.3*	16

### 3.5 Genetic context of the *mcr* variants

**Figure 3:**
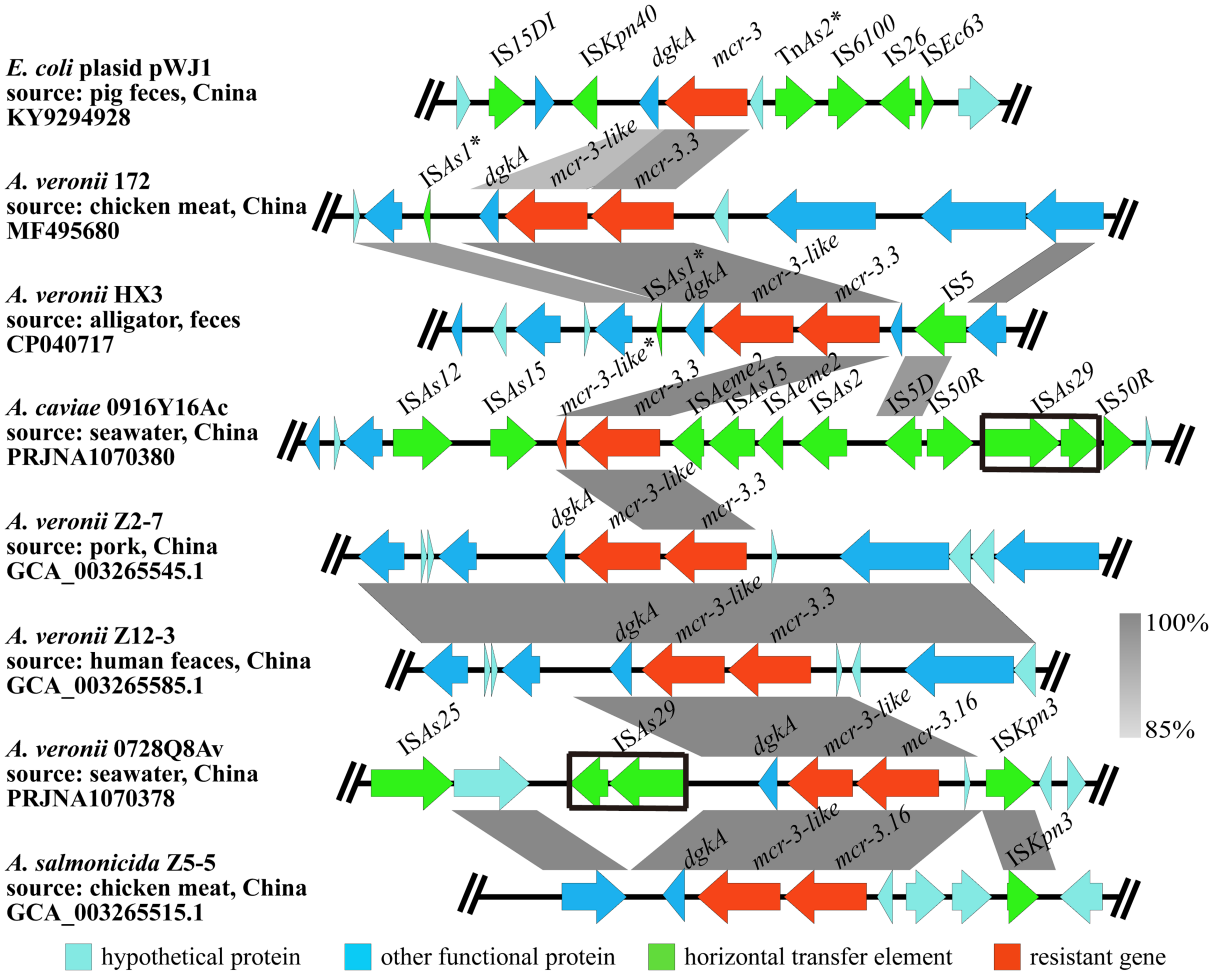
The genetic context of *mcr-3.16* and *mcr-3.3* in the *A. veronii* 0728Q8Av and *A. caviae* 1029Y16Ac, respectively. The arrows indicate the direction of gene transcription. The grayscale intensity indicates the sequence similarity between two linked regions. The black box indicates the identical insertion sequence IS*AS29*. ^*^Representing incomplete sequences.

A segment consisting of *mcr-3.16-mcr-3-like-dgkA* was observed in *A. veronii* 0728Q8Av, which was identical to that in *A. salmonicida* Z5-5 (GCA_003265515.1), a foodborne isolate from chicken meat in China. A similar genetic composition pattern *mcr-3.3-mcr-3-like-dgkA* was identified in many other *A. veronii* isolates (MF495680, CP040717, GCA_003265545.1, and GCA_003265585.1). The *mcr-3-like* gene in *A. veronii* 0728Q8Av was located 66 bp downstream of the *mcr-3.16* gene, and the *dgkA* gene was located 118 bp downstream of *mcr-3-like*, which were also found in *A. veronii* 172 (MF495680), *A. veronii* Z2-7 (GCA_003265545.1) and *A. veronii* ZJ12-3 (GCA_003265585.1). In comparison, a 66 bp intergenic region sequence existed between *mcr-3.16* (*mcr-3.3*) and *mcr-3-like* in *A. salmonicida* Z5-5 (GCA_003265515.1) and *A. veronii* HX3 (CP040717), while the intergenic region sequence between *mcr-3-like* and *dgkA* spaned 206 nucleotides. Similar to *A. salmonicida* Z5-5, the 5′ flanking region of the segment *mcr-3.16-mcr-3-like-dgkA* in *A. veronii* 0728Q8Av contained an insertion sequence IS*Kpn3*. However, in *A. salmonicida* Z5-5, two hypothetical proteins were inserted between *mcr-3.16* and IS*Kpn3*, distinguishing it from *A. veronii* 0728Q8Av. Additionally, an insertion sequence IS*As29* could be found adjacent to the *dgkA* gene in the 3′ flanking region of *mcr-3.16-mcr-3-like-dgkA* (Figure 3).

A segment consisting of *mcr-3.3-mcr-3-like* was discovered in *A. caviae* 1029Y16Ac. But the *mcr-3-like* gene was incomplete and only had 116 amino acids, which showed 100% identity to the Nitrogen terminus of the MCR-3-like (MF495680). Compared to other *Aeromonas* spp., it seemed that the 3′ end of the *mcr-3-like* and its downstream *dgkA* gene were completely absent in *A. caviae* 1029Y16Ac. None ORFs but 10 ISs, namely IS*As12*, IS*As15*, IS*As2*, IS*As29*, IS*5D*, IS*50R*, and IS*Aeme2*, were observed at the flanking regions of the segment *mcr-3.3-mcr-3-like* in *A. caviae* 1029Y16Ac (Figure 3). No transconjugants were observed on the colistin-containing screening LB agar plates coated with bacterial culture from the conjugation assay using *A. veronii* 0728Q8Av or *A. caviae* 1029Y16Ac. This indicated that the *mcr-3* variants, that is *mcr-3.16* and *mcr-3-like* in *A. veronii* 0728Q8Av and *mcr-3.3* in *A. caviae* 1029Y16Ac, were not transferred into *E. coli* J53 through conjugation, suggesting that these *mcr-3* variants were located on the chromosomes of both *A. veronii* 0728Q8Av and *A. caviae* 1029Y16Ac (Supplementary Figure S3).

### 3.6 Genetic environment of the GIs harboring MDR genes

**Figure 4:**
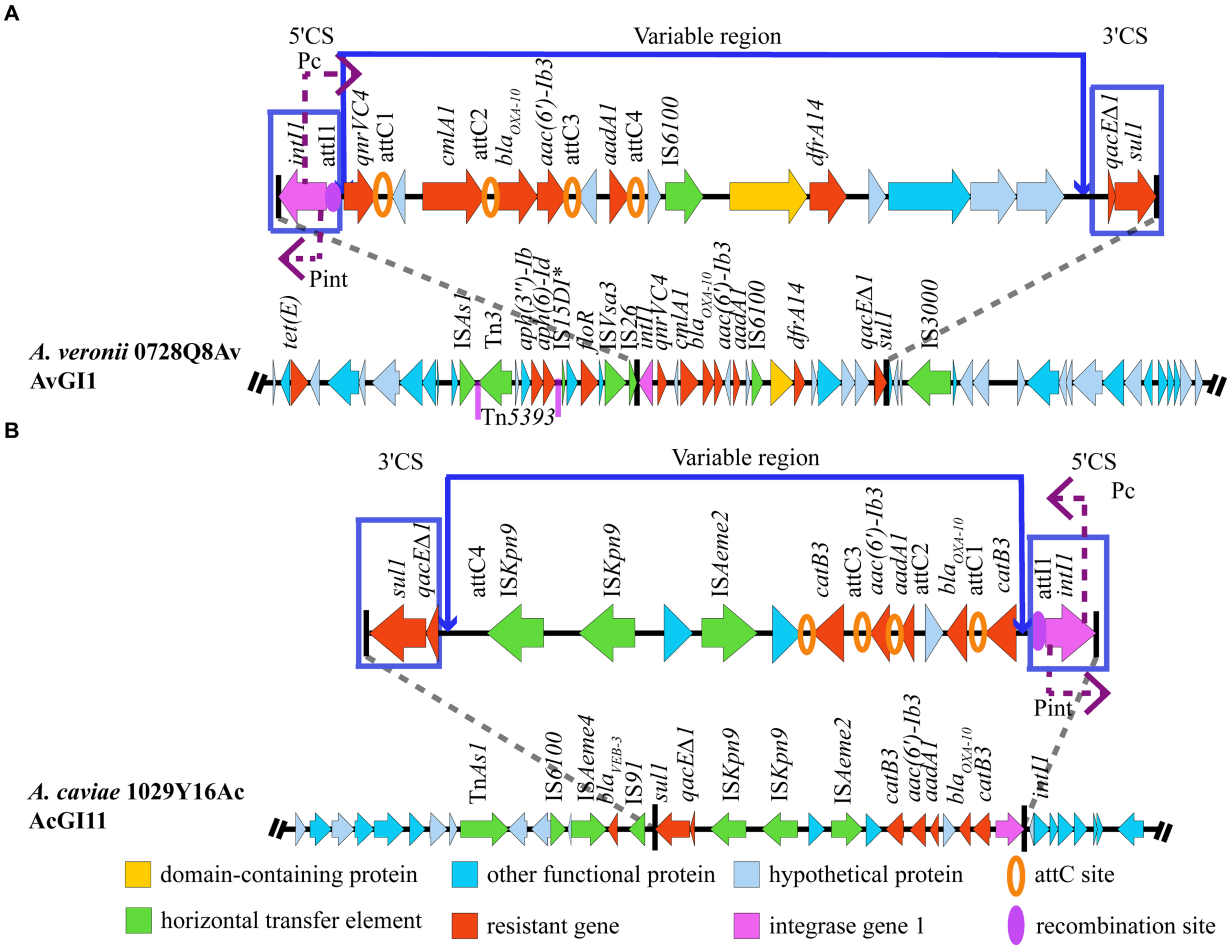
Structure of the GIs harboring MDR genes. **(A)** AvGI1 in *A. veronii* 0728Q8Av. **(B)** AcGI11 in *A. caviae* 1029Y16Ac. ^*^Representing incomplete sequences.

The results showed that gene island AvGI1 in *A. veronii* 0728Q8Av harbored 12 ARGs, including *tet(E)*, *aph (3″)-Ib*, *aph(6)-Id*, *floR*, *qnrVC4*, *cmlA1*, *bla_OXA-10_*, *aac(6′)-Ib3*, *aadA1*, *dfrA14*, truncated *qacE* (*qacE∆1*), and *sul*, which potentially mediated the resistance against tetracyclines, amphenicol, aminoglycosides, quinolones, beta lactams, quaternary ammonium compounds, and sulfonamides antibiotics (Figure 4A). Meanwhile, AvGI1 contained five complete ISs (IS*As1*, IS*Vsa3*, IS*26*, IS*6100*, and IS*3000*), one incomplete IS (IS*15DI*), and one transposon Tn*5393*. In addition, AvGI1 had a typical class 1 integron containing the integrase intI1 localized at the attI1 site and the genes *qacE∆1* and *sul1* conferring resistance to quaternary ammonium compounds and sulfonamides, respectively. The gene cassette array featured four attC sites, partitioning it into four gene boxes: *qnrVC4*, orf-*clmlA*, *bla_OXA-10_*-*aac (6′)-Ib3*, and orf-*aadA1* (Figure 4A).

The gene island AcGI11 in *A. caviae* 1029Y16Ac harbored 7 ARGs, including *bla_VEB-3_*, *sul1*, *qacE∆1*, *aac (6′)-Ib3*, *aadA1*, *bla_OXA-10_* and *catB3*, which potentially mediated the resistance against beta lactams, sulfonamides, quaternary ammonium compounds, amphenicol, and aminoglycosides (Figure 4B). Meanwhile, AcGI11 contained six complete ISs, including IS*6100*, IS*Aeme2*, IS*Aeme4*, two copies of IS*Kpn9*, and IS*91*, and one transposon Tn*As1*. Similarly, AcGI11 had the typical class 1 integron and the gene cassette array featured four attC sites, partitioning it into four gene boxes: *catB3*, *bla_OXA-10_*-orf-*aadA1*, *aac(6′)-Ib3*, and *catB3* (Figure 4B).

**Figure 5:**
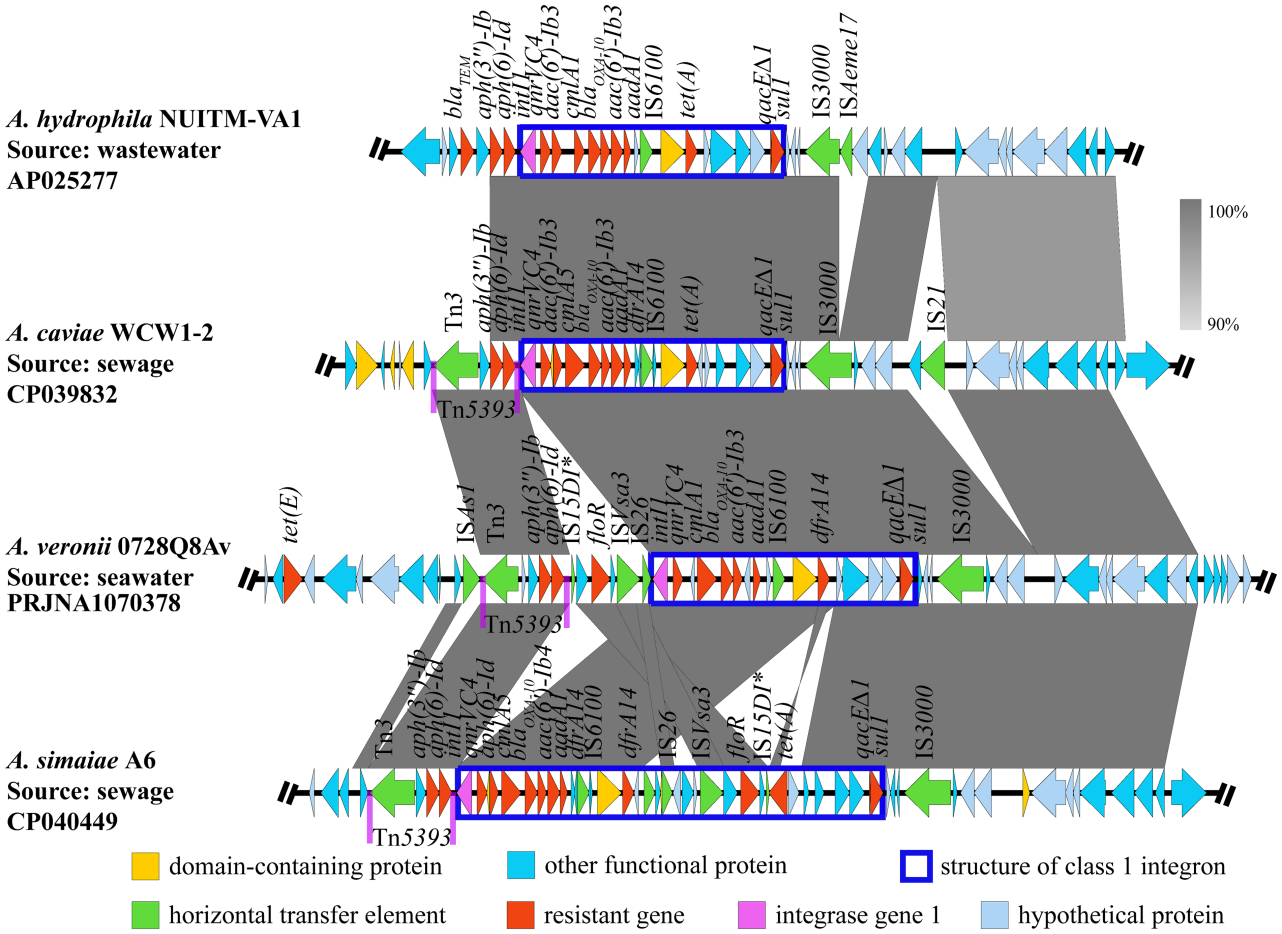
Comparative analysis of genetic environments of AvGI1. The arrows indicate the direction of gene transcription. The grayscale intensity indicates the sequence similarity between two linked regions. ^*^Representing incomplete sequences.

Blast analysis indicated that three genomic segments were similar to AvGI1 (query coverage >72%, identities >90%), that is *A. hydrophila* NUITM-VA1 (AP025277), *A. caviae* WCW1-2 (CP039832) and *A. simaiae* A6 (CP040449), while no genomic segment was similar to AcGI11. Multiple sequence alignment revealed a high similarity in the class 1 integron, including the ORFs and ISs within the gene cassettes, between *A. veronii* 0728Q8Av (PRJNA1070380) and *A. hydrophila* NUITM-VA1 (AP025277), as well as *A. caviae* WCW1-2 (CP039832). The transposon Tn*5393* and the insertion sequence IS*3000* were observed upstream and downstream of the class 1 integrons in *A. veronii* 0728Q8Av, *A. caviae* WCW1-2 (CP039832), and *A. simaiae* A6 (CP040449). But, *A. veronii* 0728Q8Av contained an additional segment, IS*15DI*-orf-*floR*-orf-IS*Vsa3*-IS*26*, located between Tn*5393* and the class 1 integron. *A. hydrophila* NUITM-VA1 (AP025277) did not form the transposon Tn*5393* (Figure 5).

### 3.7 *In vivo* bacterial pathogenicity determination

The mortality of fish infected with the *A. veronii* 0728Q8Av and *A. caviae* 1029Y16Ac isolate are summarized in Table 5. Using the calculated method reported by Mittal et al. (1980), the LD_50_ value in a 7-day period obtained from *A. veronii* 0728Q8Av was measured at 2.15 × 10^7^ CFU/mL and was categorized as virulent, while the LD_50_ of *A. caviae* 1029Y16Ac was calculated at 1.35 × 10^9^ CFU/mL and was non-virulent (Table 5).

**Table 5:** Determination of median lethal dosage (LD_50_) of *A. veronii* 0728Q8Av and *A. caviae* 1029Y16Ac.

Strain	Concentration (CFU/mL)	Sample numbers	Number of deaths at specific time (day post infection)						Mortality (%)	LD_50-7d_ (95% CI) (CFU/mL)
			1d	2d	3d	4d	6d	7d		
*A. veronii* 0728Q8Av	10^9^	30	30	0	0	0	0	0	100%	2.15 × 10^7^ (9.24 × 10^6^–4.95 × 10^7^)
	10^8^	30	11	18	1	0	0	0	100%	
	10^7^	30	0	0	2	2	1	0	16.7%	
*A. caviae* 1029Y16Ac	10^9^	30	0	0	3	2	1	0	20%	1.39 × 10^9^ (7.95 × 10^8^–2.28 × 10^9^)
	10^8^	30	0	0	2	1	0	0	10%	
	10^7^	30	0	0	1	1	0	0	6.7%	
—	PBS	30	0	0	0	0	0	0	0	—

## 4 Discussion

*Aeromonas* species isolated from coastal waters are prone to developing MDR, contributing to the interchange of ARGs between land-derived and marine environments (Zhu et al., 2017; Jang et al., 2018; Zheng et al., 2021). In this study, we pinpointed two MDR *Aeromonas* species, that is *A. veronii* 0728Q8Av and *A. caviae* 1029Y16Ac, which exhibited distinct biochemical characteristics as previously documented (Abbott et al., 2003). Both isolates exhibited resistance to colistin and harbored different *mcr-3* variants. All resistance elements, including ARGs and MGEs, were located on their chromosomes, irrespective of the presence of plasmids. The *mcr-3* variants were not clustered with other ARGs but were independently located on the chromosomal DNA, flanked by several ISs. In addition, *A. veronii* 0728Q8Av, but not *A. caviae* 1029Y16Ac, exhibits significant pathogenicity to ayu (*Plecoglossus altivelis*).

Antimicrobial resistance in *Aeromonas* is rapidly escalating worldwide, and resistance elements have become increasingly complex. For example, *Aeromonas* spp. resistance to 3rd-generation cephalosporin and producing broad-spectrum carbapenemase KPC-24 have been isolated globally (Bhaskar et al., 2015; Yang et al., 2022). *Aeromonas* demonstrates notable activity in MDR, exhibiting widespread resistance to various classes of antibiotics including β-lactam, aminoglycosides, fluoroquinolones, tetracyclines, macrolides, sulfonamides, polymyxins, and phenicols, particularly in wastewater (Figueira et al., 2011; Carusi et al., 2023; Neil et al., 2024). However, few reports have highlighted the contribution of *Aeromonas* to the development and dissemination of antimicrobial resistance elements in coastal aquatic environments (Gambino et al., 2022; Liang et al., 2024). In the present study, the two *Aeromonas* species exhibited broad MDR profiles, with sulfamethoxazole, oxytetracycline, ampicillin and florfenicol being the least effective to *A. veronii* 0728Q8Av and sulfamethoxazole and trimethoprim being the least effective to *A. caviae* 1029Y16Ac. Correspondingly, various ARGs could be found in the chromosomal genomes of both *Aeromonas* strains, such as *mcr-3.16*, *qnrVC4*, *sul1*, *dfrA14*, *bla_OXA-10_*, *ampS*, *cphA4*, *tet(E)*, *floR*, and *cmlA1* in *A. veronii* 0728Q8Av, and *mcr-3.3*, *sul1*, *bla_VEB-3_*, *bla_MOX-6_*, *bla_OXA-10_*, *tet(E)*, and *catB3* in *A. caviae* 1029Y16Ac. If only considering ARGs as determinants of antibiotic resistance, *sul1* exhibits highly potent resistance to sulfamethoxazole (with an MIC value of 256 μg/mL). No ARGs coding for trimethoprim-resistant dihydrofolate reductases (DHFRs) was found in the genome of *A. caviae* 1029Y16Ac, indicating other resistance mechanisms probably responsible for trimethoprim. Interestingly, both strains remained susceptible to ceftazidime, which is supported by their resistance gene profiles.

MGEs are the major contributors to the horizontal transfer of ARGs among bacteria (Juhas et al., 2009; Piotrowska and Popowska, 2015; shao et al., 2018). *Aeromonas* spp. can adeptly utilize these elements to develop its MDR (Piotrowska and Popowska, 2015). Unlike Enterobacteriaceae, which primarily transfer ARGs via plasmids, the extra-chromosomal DNA (Rozwandowicz et al., 2018), *Aeromonas* spp. also use plasmids for horizontal gene transfer (Piotrowska and Popowska, 2015). However, a significant proportion of their ARGs are located on the chromosome and are mainly transferred through integrons, ISs, and transposons (L’Abée-Lund and Sørum, 2001; Schmidt et al., 2001; Sakulworakan et al., 2021). ISs (e.g., IS*26*, IS*Pa12*, IS*Kpn8*,) and transposons (e.g., Tn*3*, Tn*21*, Tn*1213*) frequently involved in the formation of diverse genetic units characterized by variable regions containing various ARGs, often segmented by conservative and unique DNA structures, or forming multiple gene cassettes (Piotrowska and Popowska, 2015). Moreover, novel multiple drug-resistant MGEs are continually being identified in *Aeromonas* spp., complicating the formation and transmission of multidrug resistance further (Piotrowska and Popowska, 2015; Carusi et al., 2023). In this study, two multidrug resistance GIs, AvGI1 and AcGI11, were identified in *A. veronii* 0728Q8Av and *A. caviae* 1029Y16Ac, respectively. Both AvGI1 and AcGI11 harbored a class 1 integron which is considered the predominant type in *Aeromonas* (Piotrowska and Popowska, 2015). AvGI1 contained more ARGs than AcGI11. Apart from *sul1-qacE∆1* (a common structural combination found at 3′ end of the typical class 1 integron) (Deng et al., 2015), *aadA1*, and *bla_OXA-10_*, the ARGs in both GIs were largely distinct (Figure 4). AVGI1 contained a Tn*3* family transposase Tn*5393*, whereas AcGI11 harbored another Tn*3* family transposase Tn*As1*, and both GIs also possessed several ISs (Figure 4). Tn*5393*, located tandemly upstream of the class 1 integron in AvGI1, is likely involved in the mobility and stability of two ARGs, that is *aph (3″)-Ib* and *aph (6)-Id*, conferring resistance to aminoglycosides. Although Tn*As1*, along with ISs like IS*6100* (belonging to the IS*6* family members) and IS*91*, were found downstream of the 3′ flanking region of the integron in AcGI11, all the ARGs located within the class 1 integron. Furthermore, the ARGs were carried by the gene cassette array, and two idle IS*Kpn9*s were tandemly adjacent to the *sul1*-*qacE∆1* structure within the class 1 integron in AcGI11 (Figure 4). IS*26* was identified in the 5′ flanking region of the class 1 integron in AvGI1, forming the IS*26*-intI structure (Figure 4). However, IS*3000* was identified in the 3′ flanking region of the class 1 integron in AvGI1, forming an IS*26*-integron-IS*3000* unit rather than the IS*26*-integron-IS*26* structure reported in the literature (Shang et al., 2021; Ma et al., 2023; Wang et al., 2023). Nevertheless, the horizontal transfer of IS*26*-integron-IS*3000* remains to be explored, although IS*26*-orf-IS*3000* could serve as a vehicle for ARG transmission (Doi et al., 2014). AvGI1 exhibited high sequence similarity with several *Aeromonas* strains isolated from wastewater and sewage in China, including *A. hydrophila* NUITM-VA1 (AP025277), *A. caviae* WCW1-2 sewage (CP039832), and *A. simaiae* A6 (CP040449) (Chen C. et al., 2019; Chen Q. et al., 2019; Dao et al., 2022) (Figure 5). The composition and arrangement of ARGs carried by class 1 integron in AvGI1, together with *aph(3″)-Ib* and *aph(6)-Id*, closely resembled those observed in both *A. hydrophila* NUITM-VA1 and *A. caviae* WCW1-2. The *tet(E)* gene located not within but upstream of the class 1 integron in AvGI1, which was the only significant difference compare to *A. hydrophila* NUITM-VA1 and *A. caviae* WCW1-2. Compare to *A. hydrophila* NUITM-VA1, Tn*5393* probably enhanced the transmission of both ARGs in *A. caviae* WCW1-2 and *A. veronii* 0728Q8Av. Additionally, the Tn*5393-aph(3″)-Ib*-*aph(6)-Id* unit was also found tandemly located upstream of the class 1 integron in *A. simaiae* A6. Interestingly, an IS*15DI*-*floR*-IS*Vsa3*-IS*26* unit was also found within the class 1 integron in *A. simaiae* A6 was present as a reverse insertion between Tn*5393* and class 1 integron in AvGI1 (Figure 5). These results indicated the transposon Tn*5393*, combined with the IS IS*3000*, could serve as a vehicle for the transmission of class 1 integron, reflecting the plasticity of MGEs in ARGs transmission among bacteria.

Colistin resistance in *Aeromonas* has been extensively documented, with various *mcr* variants being detected (Ling et al., 2017; Yin et al., 2017; Eichhorn et al., 2018; Shen et al., 2018). Among these, *mcr-3* variants were detected most frequently in *Aeromonas* spp., including *mcr-3.13*, *mcr-3.14*, *mcr-3.15*, *mcr-3.16*, *mcr-3.17*, *mcr-3.18*, *mcr-3.3*, *mcr-3.6*, *mcr-3.7*, *mcr-3.8*, *mcr-3.9*, and *mcr-5* (Ling et al., 2017; Yin et al., 2017; Eichhorn et al., 2018; Ma et al., 2018; Shen et al., 2018). The *mcr-3-mcr-3-like* segment was originally reported in *A. veronni* 172 isolated from chicken meat (Ling et al., 2017). Subsequently, the *mcr-3.6-mcr-3-like*, *mcr-3.8-mcr-3-like*, and *mcr-3.9-mcr-3-like* segments were reported in *A. allosaccharophila*, *A. jandaei*, and *A. hydrophila*, respectively (Eichhorn et al., 2018). In this study, we identified a *mcr-3.16-mcr-3-like* segment in *A. veronii* 0728Q8Av and a *mcr-3.3-mcr-3-like* (incomplete) segment in *A. caviae* 1029Y16Ac. *E. coli* transformants carrying pUC19-*mcr-3.16* or pUC19-*mcr-3.16-mcr-3-like* showed an 8-fold higher colistin MIC compared to transformants containing pUC19 alone, while transformants containing pET28a-*mcr-3.16* or pET28a-*mcr-3.16-mcr-3-like* had a 2-fold higher colistin MIC than those containing pET28a alone. These results indicated that it is not *mcr-3-like* but *mcr-3.16* capable of mediating resistance to colistin in *A. veronii* 0728Q8Av, which was consistent with previous reports (Yin et al., 2017; Shen et al., 2018). In contrast, transformants carrying pUC19-*mcr-3.3* showed 4-fold higher colistin MIC than pUC19 alone, and those containing pET28a-*mcr-3.3* had a 2-fold higher colistin MIC than pET28a alone, which indicated that *mcr-3.3* could mediate resistance to colistin in *A. caviae* 1029Y16Ac (Ling et al., 2017; Shen et al., 2018). An incomplete *mcr-3-like* fragment, predictively coding partial N-terminal transmembrane domain of phosphoethanolamine transferase, was found downstream and adjacent to *mcr-3.3 A. caviae* 1029Y16Ac. This incompleteness of the phosphoethanolamine transferase mutant suggests the likelihood that *mcr-3* originated from *Aeromonas* species (Shen et al., 2018). Additionally, a *dgkA* gene encoding diacylglycerol kinase was identified downstream and adjacent to *mcr-3-like* in *A. veronii* 0728Q8Av, forming a *mcr-3.16-mcr-3-like-dgkA* segment. This positioning of *dgkA* was also found in several diploid analogs of *mcr-3* variants, such as *mcr-3.16-mcr-3-like* in *A. salmonicida* Z5-5, *mcr-3.3-mcr-3-like* in *A. veronii* Z12-3, *A. veronii* Z2-7, *A. veronii* HX3, and *A. veronii* 172 (Figure 3). Further exploration is needed to determine whether this contributes to an improvement for *mcr-3-like* in resistance to colistin. Definitely, an IS*Kpn40*-*mcr-3*-*dgkA-*IS*Kpn40* segment frequently occurred in various bacteria, such as *Klebsiella pneumoniae*, *E. coli*, and *Salmonella enterica* (Xiang et al., 2018; Hadjadj et al., 2019; Sia et al., 2020). Gallardo et al. (2020) demonstrated that *dgkA*, closely situated to *mcr-3* variants, could marginally enhance resistance to colistin. This enhancement may stem from its ability to compensate for alterations in phospholipid metabolism functions induced by LPS modification through colistin resistance determinants (Gallardo et al., 2020).

After the ban on colistin as a growth promoter in several countries for example China, Japan, and Thailand, both the colistin residue concentrations and *mcr* variants (especially *mcr-1*) in different environments have reportedly decreased (Wang et al., 2020; Rhouma et al., 2023). This has alleviated the predicament of *Aeromonas* in the spread of *mcr* genes. However, the emergence of MDR in treating *Aeromonas* infections remains a concern. In this study, the two *Aeromonas* strains exhibited similar resistance profiles, showing resistance to commonly used antibiotics such as tetracycline, oxytetracycline, florfenicol, ampicillin, sulfamethoxazole, and trimethoprim (Table 2). Both strains remained sensitive to ceftazidime and lacked common ARGs for other antibiotics, such as the *tetX* family conferring resistance to tetracycline and *bla_NDM_* conferring resistance to carbapenem, indicating that therapeutic options are still available. Nevertheless, both strains were found to carry the *bla_OXA-10_* gene encoding ESBL, and *A. caviae* 1029Y16Ac harbored the *bla_VEB-3_* gene, highlighting the need to monitor the spread of these strains.

*A. caviae* and *A. veronii*, along with *A. dhakensis*, *A. hydrophila* and *A. salmonicida*, are the most frequently detected *Aeromonas* species causing diseases in both humans and animals (Janda and Abbott, 2010; Fernández-Bravo and Figueras, 2020). As opportunistic pathogens, their pathogenicity is restricted by various factors, including genetic virulence factors, temperature, and host immune status (Fernández-Bravo and Figueras, 2020; Carusi et al., 2023). In this study, according to a virulence assessment method (Mittal et al., 1980), *A. veronii* 0728Q8Av demonstrated moderate virulence towards the tested ayu, while *A. caviae* 1029Y16Ac showed no virulence. Additionally, a 100% mortality rate was observed within 2 days post-infection when fish were injected with *A. veronii* 0728Q8Av at concentrations ranging from 10^8^ to 10^9^ CFU/mL, indicating a short incubation period for *A. veronii* 0728Q8Av infection (Chen F. et al., 2019). Compared to *A. caviae* 1029Y16Ac, more virulence factors related to pili (such as Type I, Flp type IV, and MSHA type IV) and polar flagella were identified in *A. veronii* 0728Q8Av, which probably enhanced the bacteria adhesion and persistence thus to promote its pathogenicity (Kirov et al., 2004; Boyd et al., 2008; Dacanay et al., 2010). Moreover, although the genomes of both *A. veronii* 0728Q8Av and *A. caviae* 1029Y16Ac contained genes encoding toxins hemolysin and thermostable hemolysin, only *A. veronii* 0728Q8Av additionally harbored aerolysin, a pore-forming toxin (Ran et al., 2018). This may be associated with the pathogenicity observed in *A. veronii* 0728Q8Av.

## 5 Conclusion

The MDR *Aeromonas* are ubiquitously distributed among humans, animals, and their environments. Numerous *Aeromonas* species carrying various *mcr* variants, which confer resistance to colistin, have been detected in hospitals, urban rivers, livestock, and aquatic animals. However, their presence in coastal waters remains relatively underreported. In this study, we identified two MDR *Aeromonas* strains, namely *A. veronii* 0728Q8Av and *A. caviae* 1029Y16Ac, from coastal waters in Zhejiang, China. Both *Aeromonas* isolates exhibited significant resistance to 11 kinds of antibiotics and remained susceptible to ceftazidime, a 3rd-generation cephalosporin antibiotic. And both isolates harbored multiple ARGs located on their chromosomes, with the majority concentrated within in a resistance GI, respectively. Both islands harbored typical class 1 integrons. Notably, both isolates carried ARGs mediating colistin resistance, namely *mcr-3.16* on *A. veronii* 0728Q8Av and *mcr-3.3* on *A. caviae* 1029Y16Ac. Both *mcr-3* variants were located on the chromosome, distanced from the multidrug resistance GIs, flanking by multiple ISs. Additionally, a *mcr-3-like* was identified in the genome of *A. veronii* 0728Q8Av, forming a tandem *mcr-3.16*-*mcr-3-like*-*dgkA* structure. However, the *mcr-3-like* recombinants did not confer colistin resistance in *E. coli*. Furthermore, an incomplete *mcr-3-like* was found adjacent to *mcr-3.3* in the genome of *A. caviae* 1029Y16Ac, suggesting the likelihood that *mcr-3* originated from *Aeromonas* species. Additionally, we demonstrated that *A. veronii* 0728Q8Av exhibited pathogenicity towards infected ayu. These findings indicated the presence of terrestrial MDR *Aeromonas* species in the coastal waters of China, posing a potential threat to the aquaculture, necessitating the development of more effective strategies to mitigate the spread of antibiotic resistance.

## Data availability statement

The datasets presented in this study can be found in online repositories. The names of the repository/repositories and accession number(s) can be found at: https://www.ncbi.nlm.nih.gov/genbank/, PRJNA1070378, https://www.ncbi.nlm.nih.gov/genbank/, PRJNA1070380.

## Ethics statement

The animal study was approved by the Animal Experiment Ethics Committee of Ningbo University. The study was conducted in accordance with the local legislation and institutional requirements.

## Author contributions

H-XC: Visualization, Investigation, Writing – original draft. F-JC: Investigation, Visualization, Writing – original draft. Q-JZ: Visualization, Conceptualization, Funding acquisition, Methodology, Supervision, Writing – review & editing. S-LS: Investigation, Visualization, Writing – original draft. BT: Investigation, Visualization, Writing – original draft. Z-JX: Investigation, Visualization, Writing – original draft. L-JD: Investigation, Visualization, Writing – original draft. J-LJ: Investigation, Visualization, Writing – original draft. G-ZX: Investigation, Visualization, Writing – original draft. M-CY: Resources, Writing – original draft. JC: Funding acquisition, Writing – review & editing.

## Funding

The author(s) declare that financial support was received for the research, authorship, and/or publication of this article. The work was supported by National Natural Science Foundation of China (42276110), the Program of Science and Technology Department of Zhejiang Province (LGN22C010001), the Key Research and Development Project of Zhejiang Province (2021C02059), Natural Science Foundation of Ningbo City, China (2021J061), the Program of Science and Technology Department of Ningbo City (2022S210), State Key Laboratory for Managing Biotic and Chemical Threats to the Quality and Safety of Agro-Products (2021DG700024-ZZ2102), One Health Interdisciplinary Research Project, Ningbo University (HZ202201), the Program of Zhejiang Agriculture and Rural Affairs (2023SNJF062), Zhejiang Key Laboratory of Exploitation and Preservation of Coastal Bio-Resource (J2022001).

## Footnotes

^1^ http://www.eucast.org/

^2^ http://rast.theseed.org/FIG/rast.cgi

^3^ https://cge.cbs.dtu.dk/services/ResFinder/

^4^ http://www.pathogenomics.sfu.ca/islandviewer

^5^ https://www-is.biotoul.fr/

^6^ https://tncentral.ncc.unesp.br/

^7^ https://galaxy.pasteur.fr/

^8^ https://www.ncbi.nlm.nih.gov/
