# Supplementary material for: Two colistin resistance-producing Aeromonas strains, isolated from coastal waters in Zhejiang, China: characteristics, multi-drug resistance and pathogenicity
Source: Front Microbiol. 2024 Jul 31;15:1401802. doi: 10.3389/fmicb.2024.1401802 (PMC11322120; doi:10.3389/fmicb.2024.1401802)
Supplement: Supplementary file 1 [file Data_Sheet_1.docx]

Supplementary Table 1 The primers used in this study

| **Gene name** | **Primer name** | **Primer sequence (5’-3’)** | **Product size (bp)** | **Source** |
| --- | --- | --- | --- | --- |
| *mcr-1* | *mcr-1-F* | AGTCCGTTTGTTCTTGTGGC | 320 | (Jin et al., 2021) |
|  | *mcr-1-R* | AGATCCTTGGTCTCGGCTTG |  |  |
| *mcr-2* | *mcr-2-F* | CAAGTGTGTTGGTCGCAGTT | 715 |  |
|  | *mcr-2-R* | TCTAGCCCGACAAGCATACC |  |  |
| *mcr-3* | *mcr-3-F* | AAATAAAAATTGTTCCGCTTATG | 929 |  |
|  | *mcr-3R* | AATGGAGATCCCCGTTTTT |  |  |
| *mcr-4* | *mcr-4-F* | TCACTTTCATCACTGCGTTG | 1116 |  |
|  | *mcr-4-R* | TTGGTCCATGACTACCAATG |  |  |
| *mcr-5* | *mcr-5-F* | ATGCGGTTGTCTGCATTTATC | 1644 |  |
|  | *mcr-5-R* | TCATTGTGGTTGTCCTTTTCTG |  |  |
| *mcr-6* | *mcr-6-F* | AGCTATGTCAATCCCGTGAT | 252 |  |
|  | *mcr-6-R* | ATTGGCTAGGTTGTCAATC |  |  |
| *mcr-7* | *mcr-7-F* | GCCCTTCTTTTCGTTGTT | 551 |  |
|  | *mcr-7-R* | GGTTGGTCTCTTTCTCGT |  |  |
| *mcr-8* | *mcr-8-F* | TCAACAATTCTACAAAGCGTG | 856 |  |
|  | *mcr-8-R* | AATGCTGCGCGAATGAAG |  |  |
| *mcr-9* | *mcr-9-F* | TTCCCTTTGTTCTGGTTG | 1011 |  |
|  | *mcr-9-R* | GCAGGTAATAAGTCGGTC |  |  |
| *mcr-10* | *mcr-10-F* | AGCCGTCTTGAACATGTGAG | 744 | (Xu et al., 2021) |
|  | *mcr-10-R* | CATACAGGGCACCGAGACTG |  |  |
| 16S rRNA | 16S-27F | AGAGTTTGATCCTGGCTCAG | 1400 | (Gan et al., 2020) |
|  | 16S-1492R | GGTTACCTTGTTACGACTT |  |  |
| *mcr-3.16* | Q8MCR3.16-F | CG(GGATCC)ACCTTGCAACTTTGATCCGCAC | 2685 | Designed in this study |
|  | Q8MCR3.16-R | CG(GAATTC)TCGCTGGGCGATACCTTTGTAC |  |  |
| *mcr-3-like* | MCR-3-like-F | CG(GGATCC)GGAATATGGGATGTGAAGACGGCTA | 1920 |  |
|  | MCR-3-like-R | CG(GAATTC)GAATGATTCGGGTAATTCCAGTTG |  |  |
| *mcr-3.16-mcr-3-like* | Q8MCR3.16-F | CG(GGATCC)ACCTTGCAACTTTGATCCGCAC | 4008 |  |
|  | MCR-3-like-R | CG(GGATCC)GGAATATGGGATGTGAAGACGGCTA |  |  |
| *mcr-3.3* | Y16MCR3.3-F | CG(GGATCC)AGAAATGGCCGACAATCCGC | 2787 |  |
|  | Y16MCR3.3-R | CG(GAATTC)AGCTAGGGCAATTCTAGCTCGATTA |  |  |
| ORFs of *mcr-3.16* | Q8MCR3.16-BMQ-F | CG(GAATTC)ATGCCTTCCCTTATAAAAAT | 1620 |  |
|  | Q8MCR3.16-BMQ-R | GG(CTCGAG)ATTATTCCGACATTGCTTAAAG |  |  |
| ORFs of *mcr-3-like* | Q8MCR-3-likeBMQ-F | CG(GAATTC)ATGTTTTCAGCTGTCAGAAT | 1278 |  |
|  | Q8MCR-3-likeBMQ-R | GG(CTCGAG)TCACGAAATCGGTGTAGC |  |  |
| ORFs of *mcr-3.16-mcr-3-like* | Q8MCR3.16-BMQ-F | CG(GAATTC)ATGCCTTCCCTTATAAAAAT | 2967 |  |
|  | Q8MCR-3-likeBMQ-R | GG(CTCGAG)TCACGAAATCGGTGTAGC |  |  |
| ORFs of *mcr-3.3* | Y16MCR3.3-BMQ-F | CG(GAATTC)GGAGTAAGTATGCCTTCCCTT | 1638 |  |
|  | Y16MCR3.3-BMQ-R | GG(CTCGAG)CGCAACTCAATTATTCCGAC |  |  |

Note: The base within parentheses denotes the recognition site of the restriction endonuclease. The bases at the 5' end outside parentheses function as protective bases.

**References**

Gan, C., Hu, J., Cao, Q., Zhao, R., Li, Y., Wang, Z., et al. (2020). Rapid identification of pathogens involved in pediatric osteoarticular infections by multiplex PCR. Annals of Translational Medicine, 8(5), 203. doi: 10.21037/atm.2020.01.34

Jin, J. L., Zhou, Q. J., Shao, X. B., Wang, Y. H, Chen, J., Yan, M. C., et al. (2021). Investigation on resistance genes against four types of common antibiotics in Wenzhou waters of the East China sea. *Oceanologia et limnologia Sinica*. 52, 947-959 (in Chinese). doi: 10.11693/hyhz20210200040

Xu, T., Zhang, C., Ji, Y., Song, J., Liu, Y., Guo, Y., et al. (2021). Identiﬁcation of *mcr-10* carried by self-transmissible plasmids and chromosome in *Enterobacter roggenkampii* strains isolated from hospital sewage water. *Environ. Pollut*. 268, 115706. doi: 10.1016/j.envpol.2020.115706

Supplementary Table 2 The sequences of six house-keeping genes (*gyrB*, *rpoD*, *dnaJ*, *gyrA*, *dnaX*, and *atpD*) used to construct the multilocus phylogenetic tree.

| **Strain name** | **Gene name** | **Accession number** |
| --- | --- | --- |
| *Aeromonas encheleia* strain CECT 4253-HG11 | *gyrB* | HQ442651.1 |
|  | *dnaJ* | HQ443028.1 |
|  | *gyrA* | HQ443135.1 |
|  | *dnaX* | HQ442492.1 |
|  | *atpD* | HQ442627.1 |
|  | *rpoD* | HQ442777.1 |
| *Aeromonas encheleia* strain MDC 24 | *gyrB* | HQ442652.1 |
|  | *dnaJ* | HQ443029.1 |
|  | *gyrA* | HQ443140.1 |
|  | *dnaX* | HQ442494.1 |
|  | *atpD* | HQ442625.1 |
|  | *rpoD* | HQ442775.1 |
| *Aeromonas encheleia* strain MDC 63 | *gyrB* | HQ442653.1 |
|  | *dnaJ* | HQ443027.1 |
|  | *gyrA* | HQ443136.1 |
|  | *dnaX* | HQ442497.1 |
|  | *atpD* | HQ442626.1 |
|  | *rpoD* | HQ442776.1 |
| *Aeromonas encheleia* strain MDC 64 | *gyrB* | HQ442654.1 |
|  | *dnaJ* | HQ443030.1 |
|  | *gyrA* | HQ443137.1 |
|  | *dnaX* | HQ442493.1 |
|  | *atpD* | HQ442630.1 |
|  | *rpoD* | HQ442780.1 |
| *Aeromonas encheleia* strain CECT 4342 | *gyrB* | HQ442655.1 |
|  | *dnaJ* | HQ443025.1 |
|  | *gyrA* | HQ443139.1 |
|  | *dnaX* | HQ442495.1 |
|  | *atpD* | HQ442628.1 |
|  | *rpoD* | HQ442778.1 |
| *Aeromonas encheleia* strain MDC 14 | *gyrB* | HQ442656.1 |
|  | *dnaJ* | HQ443026.1 |
|  | *gyrA* | HQ443138.1 |
|  | *dnaX* | HQ442496.1 |
|  | *atpD* | HQ442629.1 |
|  | *rpoD* | HQ442779.1 |
| *Aeromonas eucrenophila* strain CECT 4224 | *gyrB* | HQ442657.1 |
|  | *dnaJ* | HQ443015.1 |
|  | *gyrA* | HQ443115.1 |
|  | *dnaX* | HQ442509.1 |
|  | *atpD* | HQ442621.1 |
|  | *rpoD* | HQ442770.1 |
| *Aeromonas eucrenophila* strain MDC 21 | *gyrB* | HQ442658.1 |
|  | *dnaJ* | HQ443016.1 |
|  | *gyrA* | HQ443116.1 |
|  | *dnaX* | HQ442510.1 |
|  | *atpD* | HQ442620.1 |
|  | *rpoD* | HQ442771.1 |
| *Aeromonas eucrenophila* strain MDC 146 | *gyrB* | HQ442659.1 |
|  | *dnaJ* | HQ443019.1 |
|  | *gyrA* | HQ443118.1 |
|  | *dnaX* | HQ442511.1 |
|  | *atpD* | HQ442615.1 |
|  | *rpoD* | HQ442774.1 |
| *Aeromonas simiae* strain MDC 2374 | *gyrB* | GQ860942.1 |
|  | *dnaJ* | HQ443083.1 |
|  | *gyrA* | HQ443193.1 |
|  | *dnaX* | HQ442530.1 |
|  | *atpD* | HQ442645.1 |
|  | *rpoD* | GQ860943.1 |
| *Aeromonas rivuli* strain DSM 22539 | *gyrB* | FJ969434.1 |
|  | *dnaJ* | FJ969432.1 |
|  | *gyrA* | FJ969436.1 |
|  | *dnaX* | HQ442524.1 |
|  | *atpD* | HQ442641.1 |
|  | *rpoD* | FJ969433.1 |
| *Aeromonas simiae* strain CIP 107798 | *gyrB* | HQ442758.1 |
|  | *dnaJ* | HQ443081.1 |
|  | *gyrA* | HQ443191.1 |
|  | *dnaX* | HQ442528.1 |
|  | *atpD* | HQ442643.1 |
|  | *rpoD* | HQ442811.1 |
| *Aeromonas veronii* strain CECT 4257 | *gyrB* | AY101795.1 |
|  | *dnaJ* | HQ443060.1 |
|  | *gyrA* | HQ443160.1 |
|  | *dnaX* | HQ442469.1 |
|  | *atpD* | HQ442580.1 |
|  | *rpoD* | HQ442833.1 |
| *Aeromonas veronii* strain CECT 5761 | *gyrB* | HQ442729.1 |
|  | *dnaJ* | HQ443068.1 |
|  | *gyrA* | HQ443161.1 |
|  | *dnaX* | HQ442468.1 |
|  | *atpD* | HQ442583.1 |
|  | *rpoD* | HQ442831.1 |
| *Aeromonas media* strain CECT 4232 | *gyrB* | AY101782.1 |
|  | *dnaJ* | HQ443012.1 |
|  | *gyrA* | HQ443134.1 |
|  | *dnaX* | HQ442507.1 |
|  | *atpD* | HQ442614.1 |
|  | *rpoD* | HQ442785.1 |
| *Aeromonas bestiarum* strain MDC 34 | *gyrB* | HQ442684.1 |
|  | *dnaJ* | HQ442987.1 |
|  | *gyrA* | HQ443099.1 |
|  | *dnaX* | HQ442431.1 |
|  | *atpD* | HQ442544.1 |
|  | *rpoD* | HQ442857.1 |
| *Aeromonas media* strain MDC 250 | *gyrB* | HQ442707.1 |
|  | *dnaJ* | HQ443013.1 |
|  | *gyrA* | HQ443130.1 |
|  | *dnaX* | HQ442504.1 |
|  | *atpD* | HQ442613.1 |
|  | *rpoD* | HQ442781.1 |
| *Aeromonas media* strain MDC 219 | *gyrB* | HQ442705.1 |
|  | *dnaJ* | HQ443014.1 |
|  | *gyrA* | HQ443133.1 |
|  | *dnaX* | HQ442503.1 |
|  | *atpD* | HQ442612.1 |
|  | *rpoD* | HQ442784.1 |
| *Aeromonas media* strain MDC 241 | *gyrB* | HQ442706.1 |
|  | *dnaJ* | HQ443010.1 |
|  | *gyrA* | HQ443132.1 |
|  | *dnaX* | HQ442506.1 |
|  | *atpD* | HQ442610.1 |
|  | *rpoD* | HQ442783.1 |
| *Aeromonas cavernicola* strain MDC 2508 | *gyrB* | HQ442702.1 |
|  | *dnaJ* | HQ443080.1 |
|  | *gyrA* | HQ443164.1 |
|  | *dnaX* | HQ442426.1 |
|  | *atpD* | HQ442573.1 |
|  | *rpoD* | HQ442864.1 |
| *Aeromonas salmonicida* strain CECT 894 | *gyrB* | HQ442680.1 |
|  | *dnaJ* | HQ442979.1 |
|  | *gyrA* | HQ443089.1 |
|  | *dnaX* | HQ442441.1 |
|  | *atpD* | HQ442537.1 |
|  | *rpoD* | HQ442843.1 |
| *Aeromonas simiae* strain MDC 55 | *gyrB* | HQ442759.1 |
|  | *dnaJ* | HQ443082.1 |
|  | *gyrA* | HQ443192.1 |
|  | *dnaX* | HQ442529.1 |
|  | *atpD* | HQ442644.1 |
|  | *rpoD* | HQ442810.1 |
| *Aeromonas diversa* strain CECT 4254T-G501 | *gyrB* | HQ442756.1 |
|  | *dnaJ* | HQ443084.1 |
|  | *gyrA* | HQ443194.1 |
|  | *dnaX* | HQ442534.1 |
|  | *atpD* | HQ442646.1 |
|  | *rpoD* | HQ442805.1 |
| *Aeromonas diversa* strain MDC 2583 | *gyrB* | HQ442757.1 |
|  | *dnaJ* | HQ443085.1 |
|  | *gyrA* | HQ443195.1 |
|  | *dnaX* | HQ442535.1 |
|  | *atpD* | HQ442647.1 |
|  | *rpoD* | HQ442806.1 |
| *Aeromonas schubertii* strain MDC 574 | *gyrB* | HQ442753.1 |
|  | *dnaJ* | HQ443086.1 |
|  | *gyrA* | HQ443196.1 |
|  | *dnaX* | HQ442531.1 |
|  | *atpD* | HQ442650.1 |
|  | *rpoD* | HQ442753.1 |
| *Aeromonas schubertii* strain MDC 575 | *gyrB* | HQ442754.1 |
|  | *dnaJ* | HQ443087.1 |
|  | *gyrA* | HQ443197.1 |
|  | *dnaX* | HQ442532.1 |
|  | *atpD* | HQ442649.1 |
|  | *rpoD* | HQ442808.1 |
| *Aeromonas schubertii* strain CECT 4240 | *gyrB* | HQ442755.1 |
|  | *dnaJ* | HQ443088.1 |
|  | *gyrA* | HQ443198.1 |
|  | *dnaX* | HQ442533.1 |
|  | *atpD* | HQ442648.1 |
|  | *rpoD* | HQ442809.1 |
| *Aeromonas salmonicida* strain MDC 5 | *gyrB* | HQ442678.1 |
|  | *dnaJ* | HQ442982.1 |
|  | *gyrA* | HQ443090.1 |
|  | *dnaX* | HQ442442.1 |
|  | *atpD* | HQ442536.1 |
|  | *rpoD* | HQ442844.1 |
| *Aeromonas salmonicida* strain MDC 25 | *gyrB* | HQ442679.1 |
|  | *dnaJ* | HQ442980.1 |
|  | *gyrA* | HQ443091.1 |
|  | *dnaX* | HQ442443.1 |
|  | *atpD* | HQ442538.1 |
|  | *rpoD* | HQ442846.1 |
| *Aeromonas salmonicida* strain MDC 44 | *gyrB* | HQ442677.1 |
|  | *dnaJ* | HQ442981.1 |
|  | *gyrA* | HQ443092.1 |
|  | *dnaX* | HQ442444.1 |
|  | *atpD* | HQ442539.1 |
|  | *rpoD* | HQ442845.1 |
| *Aeromonas salmonicida* strain MDC 26 | *gyrB* | HQ442682.1 |
|  | *dnaJ* | HQ442983.1 |
|  | *gyrA* | HQ443093.1 |
|  | *dnaX* | HQ442445.1 |
|  | *atpD* | HQ442540.1 |
|  | *rpoD* | HQ442848.1 |
| *Aeromonas salmonicida* strain MDC 148 | *gyrB* | HQ442681.1 |
|  | *dnaJ* | HQ442984.1 |
|  | *gyrA* | HQ443094.1 |
|  | *dnaX* | HQ442446.1 |
|  | *atpD* | HQ442541.1 |
|  | *rpoD* | HQ442847.1 |
| *Aeromonas bestiarum* strain MDC 165 | *gyrB* | HQ442687.1 |
|  | *dnaJ* | HQ442985.1 |
|  | *gyrA* | HQ443095.1 |
|  | *dnaX* | HQ442427.1 |
|  | *atpD* | HQ442543.1 |
|  | *rpoD* | HQ442858.1 |
| *Aeromonas bestiarum* strain MDC 162 | *gyrB* | HQ442686.1 |
|  | *dnaJ* | HQ442989.1 |
|  | *gyrA* | HQ443096.1 |
|  | *dnaX* | HQ442428.1 |
|  | *atpD* | HQ442550.1 |
|  | *rpoD* | HQ442855.1 |
| *Aeromonas bestiarum* strain CECT 4227 | *gyrB* | HQ442683.1 |
|  | *dnaJ* | HQ442988.1 |
|  | *gyrA* | HQ443097.1 |
|  | *dnaX* | HQ442429.1 |
|  | *atpD* | HQ442556.1 |
|  | *rpoD* | HQ442854.1 |
| *Aeromonas bestiarum* strain MDC 4 | *gyrB* | HQ442685.1 |
|  | *dnaJ* | HQ442986.1 |
|  | *gyrA* | HQ443098.1 |
|  | *dnaX* | HQ442430.1 |
|  | *atpD* | HQ442542.1 |
|  | *rpoD* | HQ442856.1 |
| *Aeromonas piscicola* strain CECT 7443 | *gyrB* | HQ442690.1 |
|  | *dnaJ* | HQ442992.1 |
|  | *gyrA* | HQ443100.1 |
|  | *dnaX* | HQ442434.1 |
|  | *atpD* | HQ442545.1 |
|  | *rpoD* | HQ442859.1 |
| *Aeromonas piscicola* strain MDC 2516 | *gyrB* | HQ442692.1 |
|  | *dnaJ* | HQ442991.1 |
|  | *gyrA* | HQ443101.1 |
|  | *dnaX* | HQ442433.1 |
|  | *atpD* | HQ442547.1 |
|  | *rpoD* | HQ442860.1 |
| *Aeromonas piscicola* strain MDC 2515 | *gyrB* | HQ442689.1 |
|  | *dnaJ* | HQ442990.1 |
|  | *gyrA* | HQ443102.1 |
|  | *dnaX* | HQ442436.1 |
|  | *atpD* | HQ442549.1 |
|  | *rpoD* | HQ442862. |
| *Aeromonas piscicola* strain MDC 2518 | *gyrB* | HQ442688.1 |
|  | *dnaJ* | HQ442993.1 |
|  | *gyrA* | HQ443103.1 |
|  | *dnaX* | HQ442435.1 |
|  | *atpD* | HQ442548.1 |
|  | *rpoD* | HQ442863.1 |
| *Aeromonas piscicola* strain MDC 2517 | *gyrB* | HQ442691.1 |
|  | *dnaJ* | HQ442994.1 |
|  | *gyrA* | HQ443104.1 |
|  | *dnaX* | HQ442432.1 |
|  | *atpD* | HQ442546.1 |
|  | *rpoD* | HQ442861.1 |
| *Aeromonas popoffii* strain MDC 9 | *gyrB* | HQ442694.1 |
|  | *dnaJ* | HQ442999.1 |
|  | *gyrA* | HQ443105.1 |
|  | *dnaX* | HQ442439.1 |
|  | *atpD* | HQ442552.1 |
|  | *rpoD* | HQ442850.1 |
| *Aeromonas popoffii* strain MDC 30 | *gyrB* | HQ442695.1 |
|  | *dnaJ* | HQ442997.1 |
|  | *gyrA* | HQ443106.1 |
|  | *dnaX* | HQ442440.1 |
|  | *atpD* | HQ442553.1 |
|  | *rpoD* | HQ442849.1 |
| *Aeromonas popoffii* strain MDC 15 | *gyrB* | HQ442697.1 |
|  | *dnaJ* | HQ442996.1 |
|  | *gyrA* | HQ443107.1 |
|  | *dnaX* | HQ442475.1 |
|  | *atpD* | HQ442554.1 |
|  | *rpoD* | HQ442852.1 |
| *Aeromonas popoffii* strain CECT 5176 | *gyrB* | HQ442693.1 |
|  | *dnaJ* | HQ442995.1 |
|  | *gyrA* | HQ443108.1 |
|  | *dnaX* | HQ442437.1 |
|  | *atpD* | HQ442551.1 |
|  | *rpoD* | HQ442853.1 |
| *Aeromonas popoffii* strain MDC 23 | *gyrB* | HQ442696.1 |
|  | *dnaJ* | HQ442998.1 |
|  | *gyrA* | HQ443109.1 |
|  | *dnaX* | HQ442438.1 |
|  | *atpD* | HQ442555.1 |
|  | *rpoD* | HQ442851.1 |
| *Aeromonas molluscorum* strain CECT 5864 | *gyrB* | HQ442671.1 |
|  | *dnaJ* | HQ443000.1 |
|  | *gyrA* | HQ443110.1 |
|  | *dnaX* | HQ442519.1 |
|  | *atpD* | HQ442640.1 |
|  | *rpoD* | HQ442812.1 |
| *Aeromonas molluscorum* strain MDC 73 | *gyrB* | HQ442667.1 |
|  | *dnaJ* | HQ443002.1 |
|  | *gyrA* | HQ443111.1 |
|  | *dnaX* | HQ442521.1 |
|  | *atpD* | HQ442637.1 |
|  | *rpoD* | HQ442814.1 |
| *Aeromonas molluscorum* strain MDC 74 | *gyrB* | HQ442670.1 |
|  | *dnaJ* | HQ443001.1 |
|  | *gyrA* | HQ443112.1 |
|  | *dnaX* | HQ442522.1 |
|  | *atpD* | HQ442639.1 |
|  | *rpoD* | HQ442816.1 |
| *Aeromonas molluscorum* strain MDC 72 | *gyrB* | HQ442669.1 |
|  | *dnaJ* | HQ443004.1 |
|  | *gyrA* | HQ443114.1 |
|  | *dnaX* | HQ442520.1 |
|  | *atpD* | HQ442638.1 |
|  | *rpoD* | HQ442815.1 |
| *Aeromonas eucrenophila* strain MDC 147 | *gyrB* | HQ442660.1 |
|  | *dnaJ* | HQ443017.1 |
|  | *gyrA* | HQ443117.1 |
|  | *dnaX* | HQ442513.1 |
|  | *atpD* | HQ442616.1 |
|  | *rpoD* | HQ442772.1 |
| *Aeromonas eucrenophila* strain MDC 256 | *gyrB* | HQ442661.1 |
|  | *dnaJ* | HQ443018.1 |
|  | *gyrA* | HQ443119.1 |
|  | *dnaX* | HQ442512.1 |
|  | *atpD* | HQ442617.1 |
|  | *rpoD* | HQ442773.1 |
| *Aeromonas sp*. MDC 2473 | *gyrB* | HQ442676.1 |
|  | *dnaJ* | HQ443033.1 |
|  | *gyrA* | HQ443126.1 |
|  | *dnaX* | HQ442515.1 |
|  | *atpD* | HQ442633.1 |
|  | *rpoD* | HQ442768.1 |
| *Aeromonas sp*. MDC 2468 | *gyrB* | HQ442672.1 |
|  | *dnaJ* | HQ443032.1 |
|  | *gyrA* | HQ443127.1 |
|  | *dnaX* | HQ442514.1 |
|  | *atpD* | HQ442635.1 |
|  | *rpoD* | HQ442769.1 |
| *Aeromonas media* strain MDC 273 | *gyrB* | HQ442708.1 |
|  | *dnaJ* | HQ443011.1 |
|  | *gyrA* | HQ443131.1 |
|  | *dnaX* | HQ442505.1 |
|  | *atpD* | HQ442611.1 |
|  | *rpoD* | HQ442782.1 |
| *Aeromonas bivalvium* strain CECT 7113 | *gyrB* | HQ442703.1 |
|  | *dnaJ* | HQ443036.1 |
|  | *gyrA* | HQ443141.1 |
|  | *dnaX* | HQ442527.1 |
|  | *atpD* | HQ442557.1 |
|  | *rpoD* | HQ442817.1 |
| *Aeromonas bivalvium* strain MDC 88 | *gyrB* | HQ442704.1 |
|  | *dnaJ* | HQ443037.1 |
|  | *gyrA* | HQ443142.1 |
|  | *dnaX* | HQ442526.1 |
|  | *atpD* | HQ442558.1 |
|  | *rpoD* | HQ442818.1 |
| *Aeromonas caviae* strain MDC 51 | *gyrB* | HQ442751.1 |
|  | *dnaJ* | HQ443005.1 |
|  | *gyrA* | HQ443143.1 |
|  | *dnaX* | HQ442423.1 |
|  | *atpD* | HQ442592.1 |
|  | *rpoD* | HQ442786.1 |
| *Aeromonas caviae* strain MDC 52 | *gyrB* | HQ442752.1 |
|  | *dnaJ* | HQ443006.1 |
|  | *gyrA* | HQ443144.1 |
|  | *dnaX* | HQ442424.1 |
|  | *atpD* | HQ442593.1 |
|  | *rpoD* | HQ442787.1 |
| *Aeromonas caviae* strain MDC 33 | *gyrB* | HQ442750.1 |
|  | *dnaJ* | HQ443009.1 |
|  | *gyrA* | HQ443145.1 |
|  | *dnaX* | HQ442421.1 |
|  | *atpD* | HQ442594.1 |
|  | *rpoD* | HQ442788.1 |
| *Aeromonas caviae* strain CECT 838 | *gyrB* | HQ442748.1 |
|  | *dnaJ* | HQ443008.1 |
|  | *gyrA* | HQ443146.1 |
|  | *dnaX* | HQ442422.1 |
|  | *atpD* | HQ442602.1 |
|  | *rpoD* | HQ442790.1 |
| *Aeromonas caviae* strain MDC 49 | *gyrB* | HQ442749.1 |
|  | *dnaJ* | HQ443007.1 |
|  | *gyrA* | HQ443147.1 |
|  | *dnaX* | HQ442425.1 |
|  | *atpD* | HQ442595.1 |
|  | *rpoD* | HQ442789.1 |
| *Aeromonas sobria* strain CECT 4245 | *gyrB* | HQ442698.1 |
|  | *dnaJ* | HQ443076.1 |
|  | *gyrA* | HQ443148.1 |
|  | *dnaX* | HQ442447.1 |
|  | *atpD* | HQ442569.1 |
|  | *rpoD* | HQ442867.1 |
| *Aeromonas sobria* strain MDC 103 | *gyrB* | HQ442700.1 |
|  | *dnaJ* | HQ443078.1 |
|  | *gyrA* | HQ443149.1 |
|  | *dnaX* | HQ442449.1 |
|  | *atpD* | HQ442570.1 |
|  | *rpoD* | HQ442868.1 |
| *Aeromonas sobria* strain MDC 104 | *gyrB* | HQ442701.1 |
|  | *dnaJ* | HQ443079.1 |
|  | *gyrA* | HQ443150.1 |
|  | *dnaX* | HQ442448.1 |
|  | *atpD* | HQ442567.1 |
|  | *rpoD* | HQ442866.1 |
| *Aeromonas sobria* strain MDC 105 | *gyrB* | HQ442699.1 |
|  | *dnaJ* | HQ443077.1 |
|  | *gyrA* | HQ443151.1 |
|  | *dnaX* | HQ442450.1 |
|  | *atpD* | HQ442568.1 |
|  | *rpoD* | HQ442865.1 |
| *Aeromonas allosaccharophila* strain MDC 561 | *gyrB* | HQ442735.1 |
|  | *dnaJ* | HQ443065.1 |
|  | *gyrA* | HQ443152.1 |
|  | *dnaX* | HQ442463.1 |
|  | *atpD* | HQ442566.1 |
|  | *rpoD* | HQ442834.1 |
| *Aeromonas allosaccharophila* strain MDC 100 | *gyrB* | HQ442734.1 |
|  | *dnaJ* | HQ443064.1 |
|  | *gyrA* | HQ443153.1 |
|  | *dnaX* | HQ442460.1 |
|  | *atpD* | HQ442564.1 |
|  | *rpoD* | HQ442828.1 |
| *Aeromonas allosaccharophila* strain MDC 45 | *gyrB* | HQ442730.1 |
|  | *dnaJ* | HQ443066.1 |
|  | *gyrA* | HQ443154.1 |
|  | *dnaX* | HQ442461.1 |
|  | *atpD* | HQ442581.1 |
|  | *rpoD* | HQ442827.1 |
| *Aeromonas allosaccharophila* strain MDC 99 | *gyrB* | HQ442731.1 |
|  | *dnaJ* | HQ443063.1 |
|  | *gyrA* | HQ443155.1 |
|  | *dnaX* | HQ442459.1 |
|  | *atpD* | HQ442582.1 |
|  | *rpoD* | HQ442826.1 |
| *Aeromonas allosaccharophila* strain CECT 4199 | *gyrB* | HQ442733.1 |
|  | *dnaJ* | HQ443058.1 |
|  | *gyrA* | HQ443156.1 |
|  | *dnaX* | HQ442457.1 |
|  | *atpD* | HQ442565.1 |
|  | *rpoD* | HQ442825.1 |
| *Aeromonas veronii* strain MDC 260 | *gyrB* | HQ442726.1 |
|  | *dnaJ* | HQ443059.1 |
|  | *gyrA* | HQ443158.1 |
|  | *dnaX* | HQ442462.1 |
|  | *atpD* | HQ442571.1 |
|  | *rpoD* | HQ442830.1 |
| *Aeromonas veronii* strain MDC 181 | *gyrB* | HQ442725.1 |
|  | *dnaJ* | HQ443061.1 |
|  | *gyrA* | HQ443159.1 |
|  | *dnaX* | HQ442466.1 |
|  | *atpD* | HQ442604.1 |
|  | *rpoD* | HQ442832.1 |
| *Aeromonas veronii* strain MDC 57 | *gyrB* | HQ442727.1 |
|  | *dnaJ* | HQ443067.1 |
|  | *gyrA* | HQ443162.1 |
|  | *dnaX* | HQ442465.1 |
|  | *atpD* | HQ442584.1 |
|  | *rpoD* | HQ442835.1 |
| *Aeromonas veronii* strain CECT 4246-HG8 | *gyrB* | HQ442724.1 |
|  | *dnaJ* | HQ443069.1 |
|  | *gyrA* | HQ443163.1 |
|  | *dnaX* | HQ442467.1 |
|  | *atpD* | HQ442572.1 |
|  | *rpoD* | HQ442829.1 |
| *Aeromonas aquariorum* strain CECT 7289 | *gyrB* | HQ442712.1 |
|  | *dnaJ* | HQ443050.1 |
|  | *gyrA* | HQ443166.1 |
|  | *dnaX* | HQ442483.1 |
|  | *atpD* | HQ442590.1 |
|  | *rpoD* | HQ442798.1 |
| *Aeromonas aquariorum* strain MDC 2406 | *gyrB* | HQ442711.1 |
|  | *dnaJ* | HQ443052.1 |
|  | *gyrA* | HQ443167.1 |
|  | *dnaX* | HQ442480.1 |
|  | *atpD* | HQ442586.1 |
|  | *rpoD* | HQ442800.1 |
| *Aeromonas aquariorum* strain MDC 259 | *gyrB* | HQ442710.1 |
|  | *dnaJ* | HQ443054.1 |
|  | *gyrA* | HQ443168.1 |
|  | *dnaX* | HQ442477.1 |
|  | *atpD* | HQ442591.1 |
|  | *rpoD* | HQ442803.1 |
| *Aeromonas aquariorum* strain MDC 310 | *gyrB* | HQ442713.1 |
|  | *dnaJ* | HQ443051.1 |
|  | *gyrA* | HQ443169.1 |
|  | *dnaX* | HQ442479.1 |
|  | *atpD* | HQ442589.1 |
|  | *rpoD* | HQ442797.1 |
| *Aeromonas aquariorum* strain MDC 573 | *gyrB* | HQ442715.1 |
|  | *dnaJ* | HQ443053.1 |
|  | *gyrA* | HQ443170.1 |
|  | *dnaX* | HQ442482.1 |
|  | *atpD* | HQ442585.1 |
|  | *rpoD* | HQ442801.1 |
| *Aeromonas aquariorum* strain MDC 317 | *gyrB* | HQ442717.1 |
|  | *dnaJ* | HQ443056.1 |
|  | *gyrA* | HQ443171.1 |
|  | *dnaX* | HQ442484.1 |
|  | *atpD* | HQ442588.1 |
|  | *rpoD* | HQ442802.1 |
| *Aeromonas aquariorum* strain MDC 442 | *gyrB* | HQ442716.1 |
|  | *dnaJ* | HQ443055.1 |
|  | *gyrA* | HQ443172.1 |
|  | *dnaX* | HQ442481.1 |
|  | *atpD* | HQ442608.1 |
|  | *rpoD* | HQ442799.1 |
| *Aeromonas enteropelogenes* strain MDC 90 | *gyrB* | HQ442721.1 |
|  | *dnaJ* | HQ443042.1 |
|  | *gyrA* | HQ443173.1 |
|  | *dnaX* | HQ442486.1 |
|  | *atpD* | HQ442600.1 |
|  | *rpoD* | HQ442821.1 |
| *Aeromonas hydrophila* strain CECT 839 | *gyrB* | HQ442746.1 |
|  | *dnaJ* | HQ443048.1 |
|  | *gyrA* | HQ443174.1 |
|  | *dnaX* | HQ442472.1 |
|  | *atpD* | HQ442559.1 |
|  | *rpoD* | HQ442791.1 |
| *Aeromonas hydrophila* strain MDC 240 | *gyrB* | HQ442744.1 |
|  | *dnaJ* | HQ443049.1 |
|  | *gyrA* | HQ443175.1 |
|  | *dnaX* | HQ442470.1 |
|  | *atpD* | HQ442607.1 |
|  | *rpoD* | HQ442794.1 |
| *Aeromonas hydrophila* strain MDC 27 | *gyrB* | HQ442745.1 |
|  | *dnaJ* | HQ443044.1 |
|  | *gyrA* | HQ443176.1 |
|  | *dnaX* | HQ442476.1 |
|  | *atpD* | HQ442561.1 |
|  | *rpoD* | HQ442793.1 |
| *Aeromonas hydrophila* strain MDC 48 | *gyrB* | HQ442742.1 |
|  | *dnaJ* | HQ443045.1 |
|  | *gyrA* | HQ443177.1 |
|  | *dnaX* | HQ442474.1 |
|  | *atpD* | HQ442562.1 |
|  | *rpoD* | HQ442795.1 |
| *Aeromonas hydrophila* strain MDC 2475 | *gyrB* | HQ442743.1 |
|  | *dnaJ* | HQ443047.1 |
|  | *gyrA* | HQ443178.1 |
|  | *dnaX* | HQ442471.1 |
|  | *atpD* | HQ442560.1 |
|  | *rpoD* | HQ442792.1 |
| *Aeromonas aquariorum* strain MDC 318 | *gyrB* | HQ442714.1 |
|  | *dnaJ* | HQ443057.1 |
|  | *gyrA* | HQ443179.1 |
|  | *dnaX* | HQ442478.1 |
|  | *atpD* | HQ442587.1 |
|  | *rpoD* | HQ442804.1 |
| *Aeromonas jandaei* strain MDC 578 | *gyrB* | HQ442737.1 |
|  | *dnaJ* | HQ443070.1 |
|  | *gyrA* | HQ443184.1 |
|  | *dnaX* | HQ442451.1 |
|  | *atpD* | HQ442577.1 |
|  | *rpoD* | HQ442841.1 |
| *Aeromonas sp*. MDC 2472 | *gyrB* | HQ442673.1 |
|  | *dnaJ* | HQ443035.1 |
|  | *gyrA* | HQ443129.1 |
|  | *dnaX* | HQ442518.1 |
|  | *atpD* | HQ442632.1 |
|  | *rpoD* | HQ442766.1 |
| *Aeromonas sp*. MDC 2469 | *gyrB* | HQ442675.1 |
|  | *dnaJ* | HQ443034.1 |
|  | *gyrA* | HQ443128.1 |
|  | *dnaX* | HQ442517.1 |
|  | *atpD* | HQ442631.1 |
|  | *rpoD* | HQ442765.1 |
| *Aeromonas sp*. MDC 2467 | *gyrB* | HQ442674.1 |
|  | *dnaJ* | HQ443031.1 |
|  | *gyrA* | HQ443125.1 |
|  | *dnaX* | HQ442516.1 |
|  | *atpD* | HQ442634.1 |
|  | *rpoD* | HQ442767.1 |
| *Aeromonas tecta* strain MDC 95 | *gyrB* | HQ442666.1 |
|  | *dnaJ* | HQ443024.1 |
|  | *gyrA* | HQ443121.1 |
|  | *dnaX* | HQ442501.1 |
|  | *atpD* | HQ442619.1 |
|  | *rpoD* | HQ442761.1 |
| *Aeromonas tect*a strain MDC 94 | *gyrB* | HQ442665.1 |
|  | *dnaJ* | HQ443023.1 |
|  | *gyrA* | HQ443120.1 |
|  | *dnaX* | HQ442500.1 |
|  | *atpD* | HQ442618.1 |
|  | *rpoD* | HQ442760.1 |
| *Aeromonas tecta* strain CECT 7082 | *gyrB* | HQ442662.1 |
|  | *dnaJ* | HQ443020.1 |
|  | *gyrA* | HQ443122.1 |
|  | *dnaX* | HQ442502.1 |
|  | *atpD* | HQ442622.1 |
|  | *rpoD* | HQ442762.1 |
| *Aeromonas tecta* strain MDC 93 | *gyrB* | HQ442664.1 |
|  | *dnaJ* | HQ443021.1 |
|  | *gyrA* | HQ443124.1 |
|  | *dnaX* | HQ442499.1 |
|  | *atpD* | HQ442624.1 |
|  | *rpoD* | HQ442764.1 |
| *Aeromonas tecta* strain MDC 92 | *gyrB* | HQ442663.1 |
|  | *dnaJ* | HQ443022.1 |
|  | *gyrA* | HQ443123.1 |
|  | *dnaX* | HQ442498.1 |
|  | *atpD* | HQ442623.1 |
|  | *rpoD* | HQ442763.1 |
| *Aeromonas jandaei* strain CECT 4228 | *gyrB* | HQ442736.1 |
|  | *dnaJ* | HQ443074.1 |
|  | *gyrA* | HQ443185.1 |
|  | *dnaX* | HQ442455.1 |
|  | *atpD* | HQ442576.1 |
|  | *rpoD* | HQ442840.1 |
| *Aeromonas jandaei* strain MDC 580 | *gyrB* | HQ442740.1 |
|  | *dnaJ* | HQ443075.1 |
|  | *gyrA* | HQ443186.1 |
|  | *dnaX* | HQ442456.1 |
|  | *atpD* | HQ442578.1 |
|  | *rpoD* | HQ442839.1 |
| *Aeromonas enteropelogenes* strain CECT 4255 | *gyrB* | HQ442718.1 |
|  | *dnaJ* | HQ443038.1 |
|  | *gyrA* | HQ443187.1 |
|  | *dnaX* | HQ442490.1 |
|  | *atpD* | HQ442601.1 |
|  | *rpoD* | HQ442822.1 |
| *Aeromonas enteropelogenes* strain MDC 36 | *gyrB* | HQ442719.1 |
|  | *dnaJ* | HQ443041.1 |
|  | *gyrA* | HQ443188.1 |
|  | *dnaX* | HQ442485.1 |
|  | *atpD* | HQ442596.1 |
|  | *rpoD* | HQ442824.1 |
| *Aeromonas enteropelogenes* strain MDC 89 | *gyrB* | HQ442720.1 |
|  | *dnaJ* | HQ443043.1 |
|  | *gyrA* | HQ443189.1 |
|  | *dnaX* | HQ442487.1 |
|  | *atpD* | HQ442599.1 |
|  | *rpoD* | HQ442820.1 |
| *Aeromonas enteropelogenes* strain MDC 576 | *gyrB* | HQ442722.1 |
|  | *dnaJ* | HQ443040.1 |
|  | *gyrA* | HQ443190.1 |
|  | *dnaX* | HQ442488.1 |
|  | *atpD* | HQ442597.1 |
|  | *rpoD* | HQ442823.1 |
| *Aeromonas hydrophila* strain MDC 231 | *gyrB* | HQ442747.1 |
|  | *dnaJ* | HQ443046.1 |
|  | *gyrA* | HQ443180.1 |
|  | *dnaX* | HQ442473.1 |
|  | *atpD* | HQ442606.1 |
|  | *rpoD* | HQ442796.1 |
| *Aeromonas jandaei* strain MDC 581 | *gyrB* | HQ442739.1 |
|  | *dnaJ* | HQ443073.1 |
|  | *gyrA* | HQ443181.1 |
|  | *dnaX* | HQ442454.1 |
|  | *atpD* | HQ442575.1 |
|  | *rpoD* | HQ442838.1 |
| *Aeromonas jandaei* strain MDC 579 | *gyrB* | HQ442738.1 |
|  | *dnaJ* | HQ443072.1 |
|  | *gyrA* | HQ443182.1 |
|  | *dnaX* | HQ442453.1 |
|  | *atpD* | HQ442574.1 |
|  | *rpoD* | HQ442837.1 |
| *Aeromonas jandaei* strain MDC 582 | *gyrB* | HQ442741.1 |
|  | *dnaJ* | HQ443071.1 |
|  | *gyrA* | HQ443183.1 |
|  | *dnaX* | HQ442452.1 |
|  | *atpD* | HQ442579.1 |
|  | *rpoD* | HQ442842.1 |
| *Aeromonas enteropelogenes* strain MDC 577 | *gyrB* | HQ442723.1 |
|  | *dnaJ* | HQ443039.1 |
|  | *gyrA* | HQ443165.1 |
|  | *dnaX* | HQ442489.1 |
|  | *atpD* | HQ442598.1 |
|  | *rpoD* | HQ442819.1 |
| *Aeromonas caviae* strain WCW1-2 | *gyrB* | QDO73926.1 |
|  | *dnaJ* | QDO76504.1 |
|  | *gyrA* | QDO75897.1 |
|  | *dnaX* | QDO75774.1 |
|  | *atpD* | QDO77684.1 |
|  | *rpoD* | QDO74553.1 |
| *Aeromonas hydrophila* NUITM-VA1 | *gyrB* | BDC80061.1 |
|  | *dnaJ* | BDC80677.1 |
|  | *gyrA* | BDC82514.1 |
|  | *dnaX* | BDC82376.1 |
|  | *atpD* | BDC84601.1 |
|  | *rpoD* | BDC83824.1 |
| *Aeromonas simiae* strain A6 | *gyrB* | QFI56335.1 |
|  | *dnaJ* | QFI54110.1 |
|  | *gyrA* | QFI54736.1 |
|  | *dnaX* | QFI55064.1 |
|  | *atpD* | QFI56379.1 |
|  | *rpoD* | QFI55929.1 |
| *Aeromonas veronii* strain HX3 | *gyrB* | QGW97546.1 |
|  | *dnaJ* | QGW96303.1 |
|  | *gyrA* | QGW95742.1 |
|  | *dnaX* | QGW95508.1 |
|  | *atpD* | QGW97519.1 |
|  | *rpoD* | QGW96908.1 |
| *Aeromonas veronii* strain FC951 | *gyrB* | AYK16494.1 |
|  | *dnaJ* | AYK17612.1 |
|  | *gyrA* | AYK18257.1 |
|  | *dnaX* | AYK17990.1 |
|  | *atpD* | AYK20090.1 |
|  | *rpoD* | YP_855378.1 |
| *Aeromonas veronii* strain ZfB1 | *gyrB* | QIF42562.1 |
|  | *dnaJ* | QIF43791.1 |
|  | *gyrA* | QIF44553.1 |
|  | *dnaX* | QIF44233.1 |
|  | *atpD* | QIF46408.1 |
|  | *rpoD* | QIF46014.1 |
| *Aeromonas veronii* strain X12 | *gyrB* | ATY79397.1 |
|  | *dnaJ* | ATY81895.1 |
|  | *gyrA* | ATY81047.1 |
|  | *dnaX* | ATY80925.1 |
|  | *atpD* | ATY83280.1 |
|  | *rpoD* | ATY82508.1 |
| *Aeromonas caviae* GSH8M-1 | *gyrB* | BBG87338.1 |
|  | *dnaJ* | BBG90255.1 |
|  | *gyrA* | BBG89526.1 |
|  | *dnaX* | BBG89407.1 |
|  | *atpD* | BBG91515.1 |
|  | *rpoD* | BBG90820.1 |
| *Aeromonas hydrophila* strain B11 | *gyrB* | QKF97670.1 |
|  | *dnaJ* | QKG00412.1 |
|  | *gyrA* | QKF99608.1 |
|  | *dnaX* | QKF99731.1 |
|  | *atpD* | QKG01648.1 |
|  | *rpoD* | QKF98403.1 |
| *Aeromonas sp.* ASNIH2 | *gyrB* | AUY09876.1 |
|  | *dnaJ* | AUY08701.1 |
|  | *gyrA* | AUY08155.1 |
|  | *dnaX* | AUY11846.1 |
|  | *atpD* | AUY09902.1 |
|  | *rpoD* | AUY09257.1 |
| *Aeromonas molluscorum* strain MDC 43 | *gyrB* | HQ442668.1 |
|  | *dnaJ* | HQ443003.1 |
|  | *gyrA* | HQ443113.1 |
|  | *dnaX* | HQ442523.1 |
|  | *atpD* | HQ442636.1 |
|  | *rpoD* | HQ442813.1 |

Supplementary Table 3 Prediction results of genomic island of *A. veronii* 0728Q8Av and *A. caviae* 1029Y16Ac

| **Gene island number** | **Gene island initiation site** | **Gene island termination site** | **Gene island length** |
| --- | --- | --- | --- |
| **AvGI1** | **474481** | **534909** | **60428** |
| AvGI2 | 657804 | 667485 | 9681 |
| AvGI3 | 825279 | 853343 | 28064 |
| AvGI4 | 1505541 | 1575830 | 70289 |
| AvGI5 | 1964457 | 1978575 | 14118 |
| AvGI6 | 2024712 | 2033285 | 8573 |
| AvGI7 | 2254125 | 2258922 | 4797 |
| AvGI8 | 2558050 | 2566978 | 8928 |
| AvGI9 | 2807425 | 2820818 | 13393 |
| AvGI10 | 2894428 | 2913396 | 18968 |
| AvGI11 | 3095616 | 3120435 | 24819 |
| AvGI12 | 3272545 | 3284547 | 12002 |
| AvGI13 | 3574865 | 3600184 | 25319 |
| AvGI14 | 3658843 | 3699255 | 40412 |
| AvGI15 | 3958657 | 3973202 | 14545 |
| AcGI1 | 322951 | 383501 | 60550 |
| AcGI2 | 561171 | 566792 | 5621 |
| AcGI3 | 570588 | 580444 | 9856 |
| AcGI4 | 955648 | 964505 | 8857 |
| AcGI5 | 1558081 | 1569307 | 11226 |
| AcGI6 | 1691795 | 1700260 | 8465 |
| AcGI7 | 1944125 | 1975683 | 31558 |
| AcGI8 | 2162059 | 2172923 | 10864 |
| AcGI9 | 2278007 | 2282878 | 4871 |
| AcGI10 | 2317932 | 2324029 | 6097 |
| **AcGI11** | **2378406** | **2410254** | **31848** |
| AcGI12 | 2474770 | 2498738 | 23968 |
| AcGI13 | 2546007 | 2586018 | 40011 |
| AcGI14 | 2700948 | 2711969 | 11021 |
| AcGI15 | 2935645 | 2939492 | 3847 |
| AcGI16 | 2973361 | 2978182 | 4821 |
| AcGI17 | 3010110 | 3041998 | 31888 |
| AcGI18 | 3230902 | 3238946 | 8044 |
| AcGI19 | 3359155 | 3386448 | 27293 |
| AcGI20 | 3667177 | 3689454 | 22277 |
| AcGI21 | 3776630 | 3805872 | 29242 |
| AcGI22 | 4365177 | 4370211 | 5034 |
| AcGI23 | 4785891 | 4805546 | 19655 |

Note: The AvGI1 and AcGI11 highlighted in bold represent multidrug resistance genomic islands.


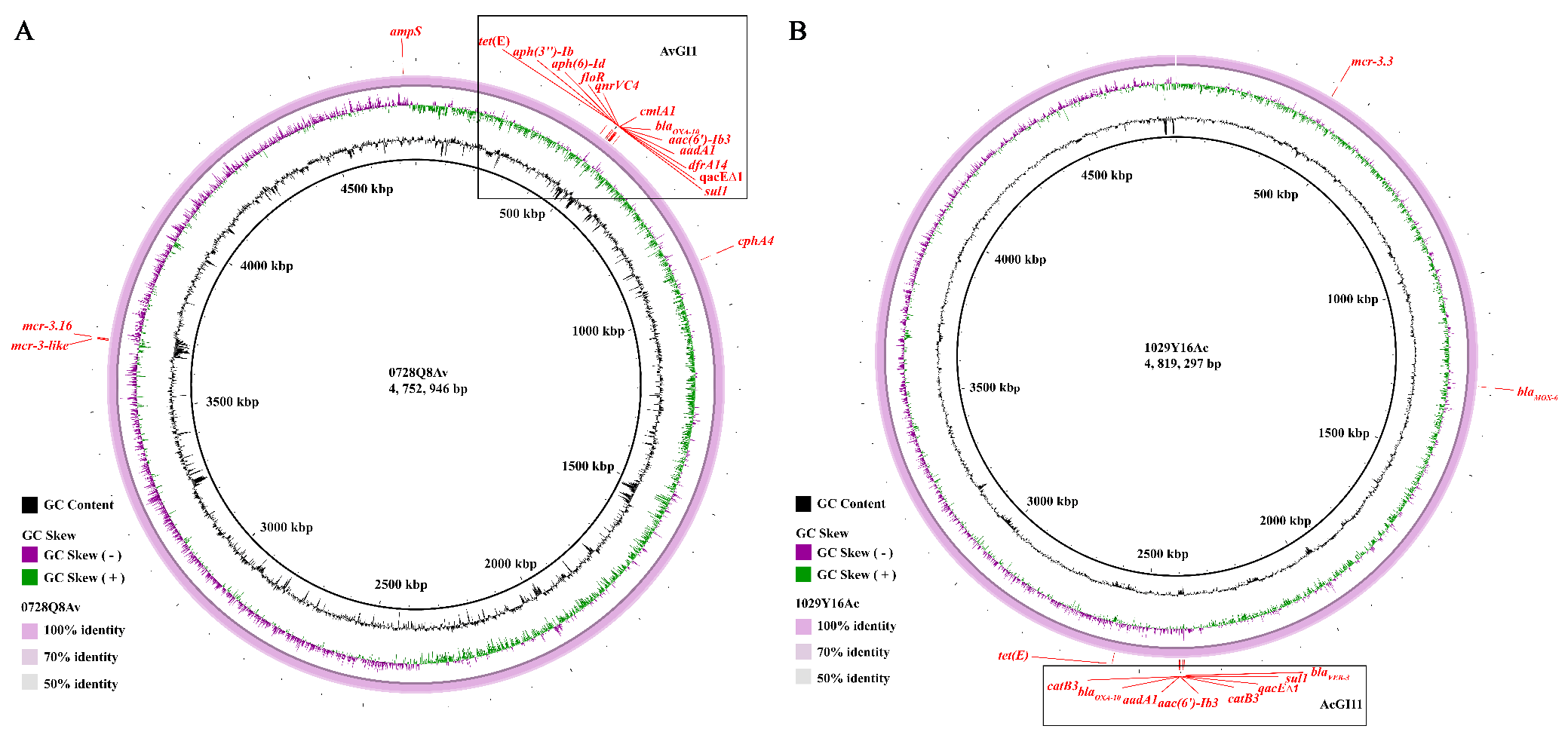


Supplementary Figure 1. Genetic location of antibiotic resistance genes in the genomes of *A. veronii* 0728Q8Av and *A. caviae* 1029Y16Ac. A, *A. veronii* 0728Q8Av. B, *A. caviae* 1029Y16Ac. It includes, from inner to outer rings, the nucleotide sequence of the strain, GC content, GC skew, genome of strain and marked resistance genes.


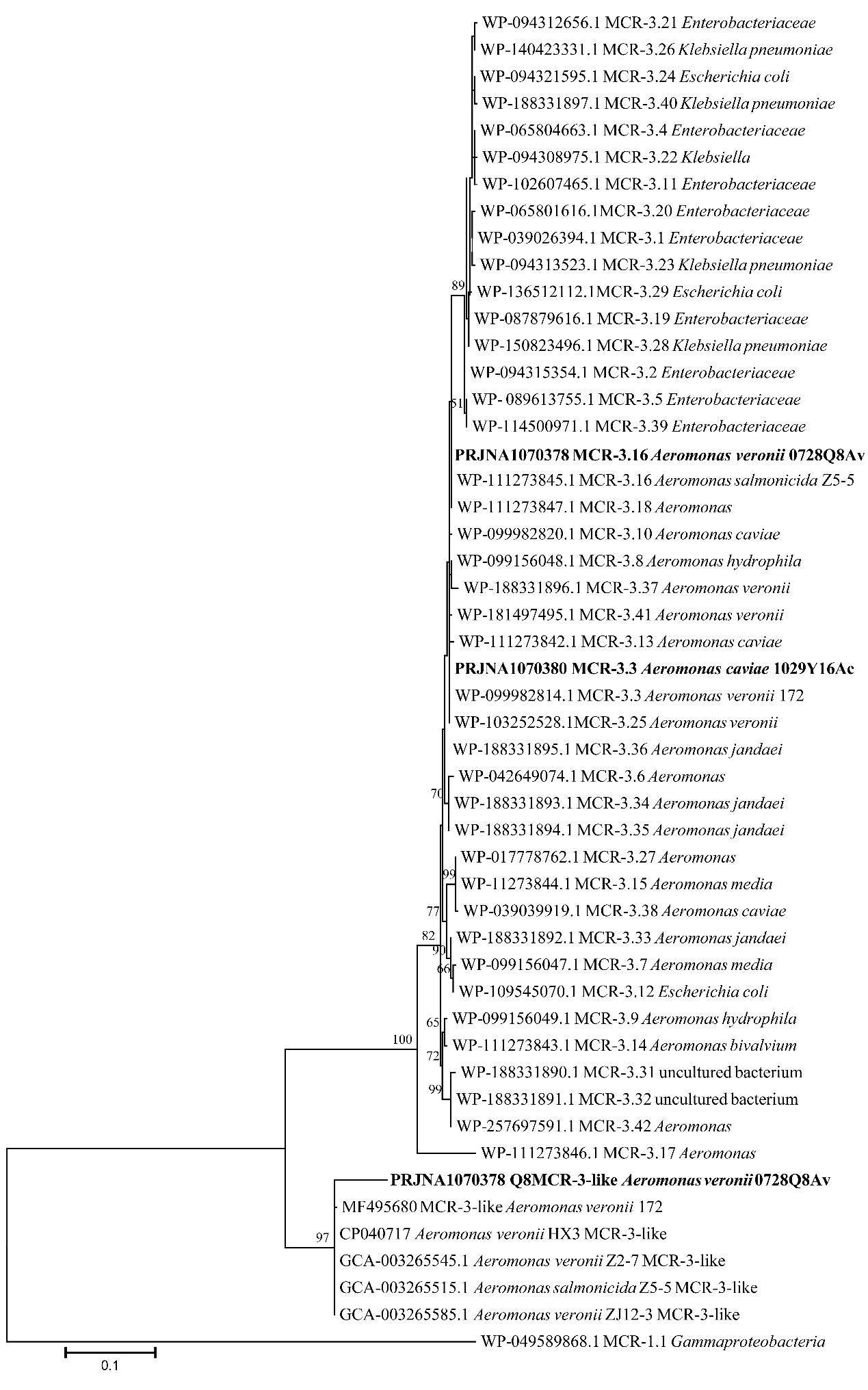


Supplementary Figure 2 Phylogenetic analysis of the MCR-3 sequences. Phylogenetic tree of the deduced amino acid sequences of putative phosphoethanolaminen transferases from different bacterial species. The tree was generated using MEGA 7 by maximum likelihood method.


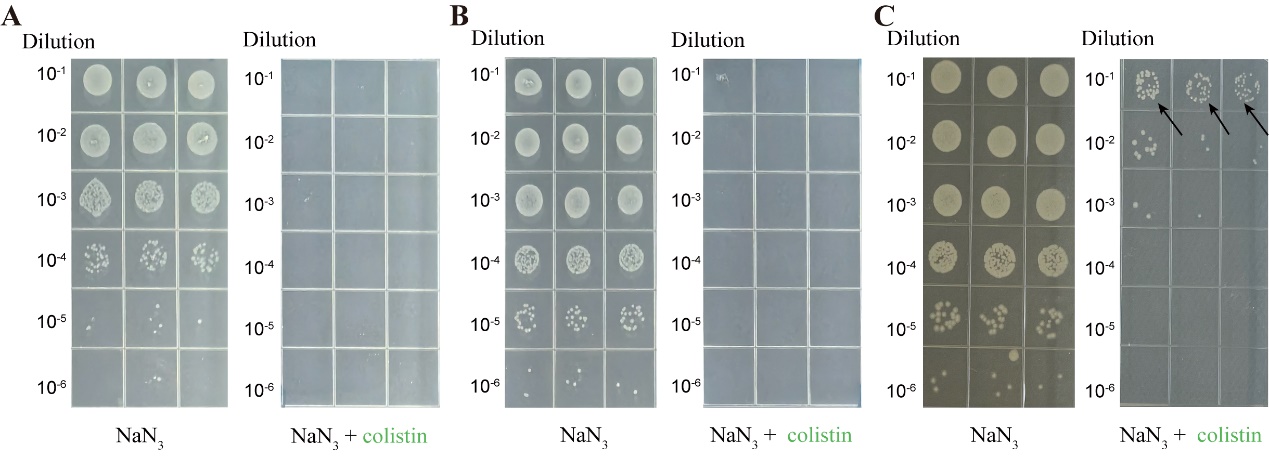


Supplementary Figure 3 Conjugation assays using *A. veronii* 0728Q8Av and *A. caviae* 1029Y16Ac as the donor strain, respectively. Growth phenotypes of transconjugants on LB agar plates containing colistin + NaN_3_ and NaN_3_ using different dilutions of bacterial culture, respectively were illustrated as follows: The *E. coli* J53 was used as the recipient strain; and (A) *A. veronii 0728Q8Av* was the donor strain; and (B) *A. caviae* 1029Y16Ac was the donor strain; and (C) The *E. coli* ECCNB20-2 carrying *mcr-1* was used as a positive control donor. The bacteria via conjugation were transferred to filter paper for 8 h-culture and the bacterial culture was suspended into LB broth, and then diluted into 10^-1^, 10^-2^, 10^-3^, 10^-4^, 10^-5^, and 10^-6^.
